# CHK1 protects oncogenic KRAS-expressing cells from DNA damage and is a target for pancreatic cancer treatment

**DOI:** 10.1016/j.celrep.2021.110060

**Published:** 2021-11-30

**Authors:** Jennifer E. Klomp, Ye S. Lee, Craig M. Goodwin, Björn Papke, Jeff A. Klomp, Andrew M. Waters, Clint A. Stalnecker, Jonathan M. DeLiberty, Kristina Drizyte-Miller, Runying Yang, J. Nathaniel Diehl, Hongwei H. Yin, Mariaelena Pierobon, Elisa Baldelli, Meagan B. Ryan, Siqi Li, Jackson Peterson, Amber R. Smith, James T. Neal, Aaron K. McCormick, Calvin J. Kuo, Christopher M. Counter, Emanuel F. Petricoin, Adrienne D. Cox, Kirsten L. Bryant, Channing J. Der

**Affiliations:** 1 Lineberger Comprehensive Cancer Center, University of North Carolina at Chapel Hill, Chapel Hill, NC 27599, USA; 2 Department of Pharmacology, University of North Carolina at Chapel Hill, Chapel Hill, NC 27599, USA; 3 Curriculum in Genetics and Molecular Biology, University of North Carolina at Chapel Hill, Chapel Hill, NC 27599, USA; 4 Departments of Cancer and Cell Biology, Translational Genomics Research Institute, Phoenix, AZ, USA; 5 Center for Applied Proteomics and Molecular Medicine, George Mason University, Manassas, VA 20110, USA; 6 Department of Pharmacology and Cancer Biology, Duke University Medical Center, Durham, NC; 7 Department of Medicine, Stanford University, Stanford University School of Medicine, Stanford, CA 94305, USA; 8 Department of Radiation Oncology, University of North Carolina at Chapel Hill, Chapel Hill, NC 27599, USA; 9 Lead contact

## Abstract

We apply genetic screens to delineate modulators of KRAS mutant pancreatic ductal adenocarcinoma (PDAC) sensitivity to ERK inhibitor treatment, and we identify components of the ATR-CHK1 DNA damage repair (DDR) pathway. Pharmacologic inhibition of CHK1 alone causes apoptotic growth suppression of both PDAC cell lines and organoids, which correlates with loss of MYC expression. CHK1 inhibition also activates ERK and AMPK and increases autophagy, providing a mechanistic basis for increased efficacy of concurrent CHK1 and ERK inhibition and/or autophagy inhibition with chloroquine. To assess how CHK1 inhibition-induced ERK activation promotes PDAC survival, we perform a CRISPR-Cas9 loss-of-function screen targeting direct/indirect ERK substrates and identify RIF1. A key component of non-homologous end joining repair, RIF1 suppression sensitizes PDAC cells to CHK1 inhibition-mediated apoptotic growth suppression. Furthermore, ERK inhibition alone decreases RIF1 expression and phenocopies RIF1 depletion. We conclude that concurrent DDR suppression enhances the efficacy of ERK and/or autophagy inhibitors in KRAS mutant PDAC.

## In brief

Klomp et al. show that CHK1 is essential for KRAS mutant pancreatic cancer cell growth. CHK1 inhibition causes apoptotic growth suppression, MYC loss, and compensatory ERK and autophagy activation. Concurrent CHK1, ERK, and/or autophagy inhibition enhances apoptotic growth suppression. Additionally, genetic depletion of ERK-regulated DNA damage repair protein RIF1 phenocopies ERK inhibition.

## Graphical Abstract

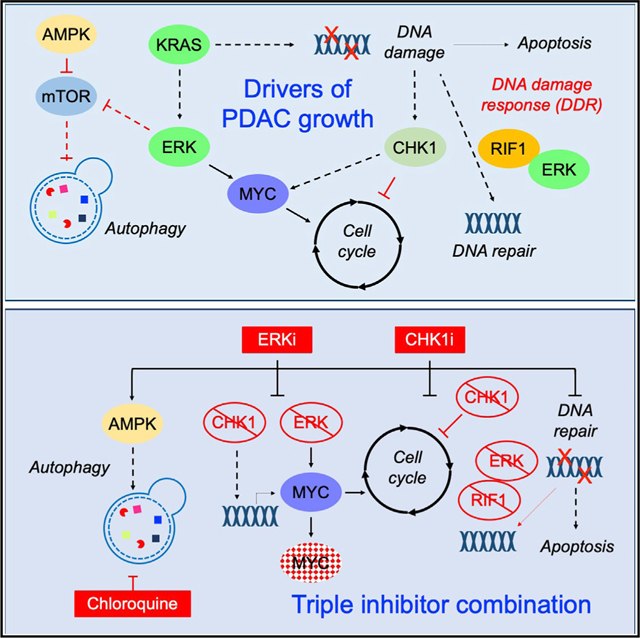

## INTRODUCTION

Pancreatic ductal adenocarcinoma (PDAC) is the third leading cause of cancer-related deaths in the United States, with a dismal 5-year survival rate of 10% (Siegel et al., 2021). Despite the well-defined genetic landscape of PDAC (Waters and Der, 2018), to date no clinically effective targeted therapies have been developed, and current standards of care remain conventional cytotoxic drugs. Mutations in the *KRAS* oncogene occur in >95% of cases, and the role of mutant KRAS in driving PDAC growth is well established. Although progress has been made in the clinical development of direct inhibitors of one KRAS mutation (G12C), this mutation constitutes only 2% of KRAS mutations in PDAC (Moore et al., 2020; Ryan and Corcoran, 2018). Therefore, indirect approaches remain the best strategies for targeting the majority of KRAS mutant PDAC (Papke and Der, 2017).

Inhibitors of KRAS effector signaling networks are the most promising indirect strategy to target mutant KRAS function for cancer treatment. Among the multitude of downstream effectors, the RAF-MEK-ERK mitogen-activated protein kinase (MAPK) cascade is one of the most intensively pursued target for blocking KRAS oncogenic activity. Potent and selective inhibitors of each node of this protein kinase cascade have been developed (Moore et al., 2020; Papke and Der, 2017; Ryan and Corcoran, 2018). However, the clinical efficacy of these inhibitors as monotherapy has been limited by cancer cell resistance and normal cell toxicity (Bennouna et al., 2011; Bodoky et al., 2012; Hainsworth et al., 2010). Cancer cell resistance is mediated, in part, by treatment-induced loss of ERK-dependent negative feedback and the resulting upstream reactivation of RAF-MEK-ERK signaling (Klomp et al., 2021; Lake et al., 2016). Strategies to overcome resistance to ERK MAPK pathway inhibitors include the application of unbiased genetic and chemical library screens to identify effective drug combinations (Corcoran et al., 2013; Lito et al., 2014; Sulahian et al., 2019; Ozkan-Dagliyan et al., 2020). For example, we and others identified combinations of MEK/ERK inhibition (MEKi/ERKi) together with autophagy inhibition for KRAS mutant PDAC (Bryant et al., 2019; Kinsey et al., 2019), providing the rationale for our initiation of clinical trials evaluating the combinations of MEKi/ERKi and hydroxychloroquine in this disease (NCT04132505, NCT04386057).

Another indirect anti-RAS strategy involves targeting the DNA damage response (DDR) that allows cancer cells to counteract the lethal consequences of oncogenic KRAS-induced replicative stress and genomic instability (Luo et al., 2009; Grabocka et al., 2015; Primo and Teixeira, 2019). The DDR is initiated by the sensing of DNA single-strand breaks (SSBs) or double-strand breaks (DSBs), resulting in activation of the ATR-CHK1 or ATM-CHK2 kinase signaling networks, respectively. These networks impair G_2_/M cell cycle progression through inhibition of cyclin-dependent kinases, which allows time for DNA repair and prevents the accumulation of toxic DNA damage. Thus, inhibitors of DDR-promoting kinases may preferentially target the growth of KRAS mutant cancer cells by allowing G_2_/M progression to proceed in the presence of unrepaired DNA damage. Such inhibitors may be even more effective in combination with DNA-damaging agents that induce the DDR. In particular, as a driver role for ATR-CHK1 in supporting cancer growth has been described (Forment and O’Connor, 2018; Qiu et al., 2018; Smith et al., 2010), inhibitors of this kinase axis have been developed, primarily targeting CHK1 (Dent, 2019; Qiu et al., 2018). Supporting the potential therapeutic value of targeting CHK1 in PDAC, co-treatment with CHK1-selective inhibitors sensitized PDAC cells to gemcitabine and/or radiation in preclinical models (Engelke et al., 2013; Morgan et al., 2010; Parsels et al., 2011). However, although numerous CHK1 inhibitors have advanced to clinical evaluation (Qiu et al., 2018), treatment of PDAC patients with CHK1i and gemcitabine did not show a clinical benefit over gemcitabine alone (Laquente et al., 2017). Thus, the therapeutic value of targeting the DDR in pancreatic cancer remains unresolved.

In the present study, we applied genetic screens and determined that CHK1 loss promotes the anti-proliferative activity of ERK inhibitors in KRAS mutant PDAC. We determined that CHK1 inhibition also caused upregulation of autophagic flux and increased phosphorylated ERK (pERK), both likely compensation mechanisms to promote cell survival. We found that dual inhibition of CHK1 and autophagy led to significant increases in growth suppression and apoptosis and that these effects were further enhanced upon ERK inhibition. We conclude that ERK regulation of the DDR is an important output of this key KRAS effector signaling network.

## RESULTS

### DDR genes modulate sensitivity to ERK inhibition

**Figure 1.**
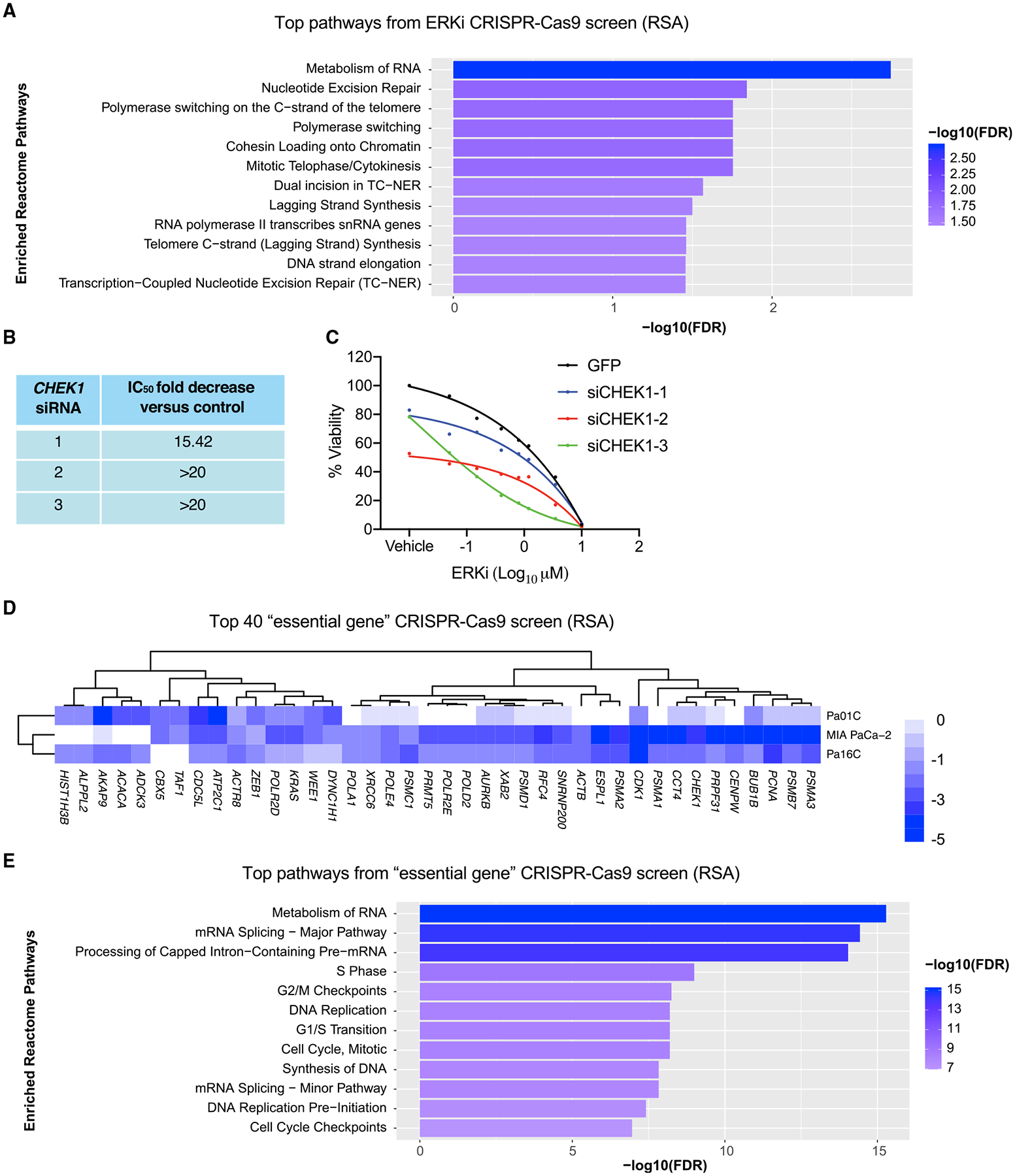
CHEK1 is identified as an ERKi sensitizer and essential for PDAC growth (A) Pathway analysis of the top 50 genes identified in a loss-of-function CRISPR-Cas9 screen (Pa01C, Pa14C, and PANC-1) targeting the druggable genome. Enriched ERKi sensitizer reactomes were determined using a STRING false discovery rate set at 5% and comparing the mean logP of all cell types and time points of the entire library. (B) The shifts in ERKi GI_50_ with the top three *CHEK1* siRNAs from the initial siRNA druggable genome screen. (C) Viability curves (Pa16C) following 4 day treatment with ERKi and 5 day treatment with *CHEK1* siRNA, with GFP siRNA as a control. (D) The top 40 genes from the CRISPR-Cas9 “druggable genome” viability screen for identification of genes essential to PDAC cell growth; scale references the logP (RSA) value. (E) Reactomes enriched with “essential” genes were identified using a STRING false discovery rate of 5% as described in (A).

We showed previously that a subset of KRAS mutant PDAC cell lines exhibited sensitivity to ERK1/2-selective inhibitors (Figure S1A) (Hayes et al., 2016). To identify drug combinations that enhance the sensitivity of *KRAS* mutant PDAC cell lines to the ERK inhibitor SCH772984 (ERKi), we applied both CRISPR-Cas9 and small interfering RNA (siRNA) genetic loss-of-function screens targeting genes that constitute the druggable genome (Figures S1B and S1C). As expected, *KRAS* was one of the strongest hits in the CRISPR screen (Figure S1D). Pathway analysis of the top 50 hits identified genes involved in several aspects of the DDR (e.g., *ATR*) (Figure 1A). In the siRNA screen, 38 genes were identified for which two or more siRNAs caused at least a 5-fold decrease in the ERKi GI_50_ (50% of maximal inhibition of cell proliferation/growth) relative to the control (Waters et al., 2021). Among these was *CHEK1*, which encodes CHK1, a key component of the ATR-CHK1 DDR pathway. siRNA suppression of *CHEK1* caused a greater than 15-fold decrease in the ERKi GI_50_ (Figure 1B), and this activity was validated in a secondary screen (Figure 1C).

To discern which of the ERKi sensitizer genes were also essential for cell viability, we used the same barcoded CRISPR library (Figure S1B) to determine which genes dropped out at 9 days post-library infection. As expected, *KRAS* was among the top 40 genes identified as essential for viability (Figure 1D). *CHEK1* was also among these top hits (Figure 1D). Kinome data from the Cancer Dependency Map (DepMap) CRISPR (Figure S1E) and shRNA (Figure S1F) screens also support PDAC growth dependency on *CHEK1* and *ATR* expression. We also determined that siRNA-mediated genetic suppression of *CHEK1* expression significantly suppressed growth, comparable with that seen with *KRAS* suppression (Figure S1G). Depletion of *ATM* or *ATR* also decreased growth, albeit to a lesser degree than *CHEK1*. Additionally, whereas depletion of *ATM* or *ATR* did not cause significant formation of γH2AX, a marker of double-stranded (ds) DNA breaks, *CHEK1* suppression strongly induced γH2AX (Figure S1H). Finally, Kaplan-Meier analyses of RNA sequencing (RNA-seq) data from The Cancer Genome Atlas (TCGA) demonstrated that high expression of *CHEK1*, but not *ATM*, *ATR*, or *CHEK2*, correlated with poor survival (Figures S1I and S1J). Collectively, these data support a therapeutic potential of targeting CHK1 in KRAS mutant PDAC. We focused on elucidating a mechanistic basis whereby concurrent CHK1 inhibition may enhance ERK inhibitor-mediated suppression of PDAC growth.

### CHK1 inhibition causes apoptotic growth suppression

**Figure 2.**
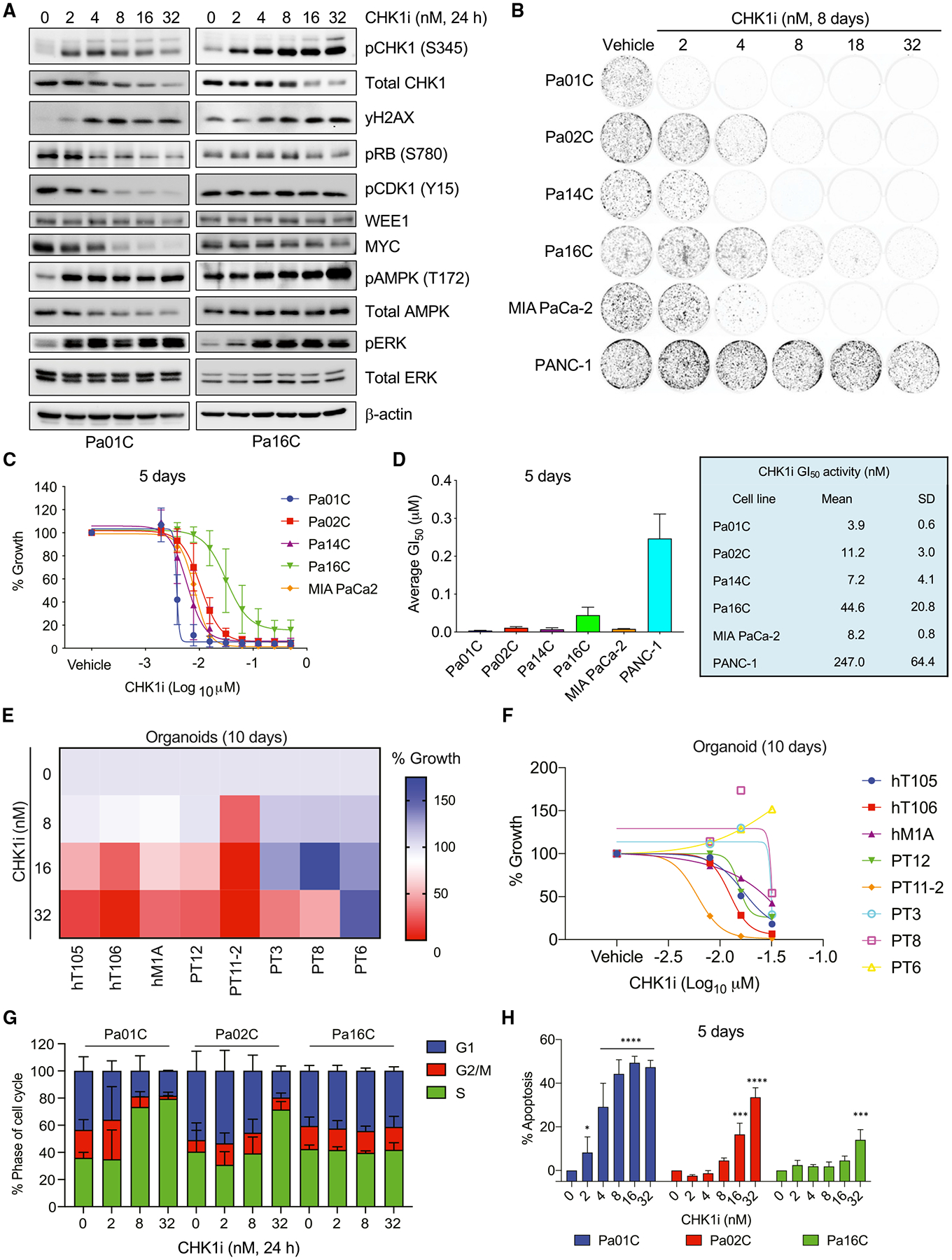
CHK1i blocks PDAC growth and induces S-phase arrest and apoptosis (A) Immunoblot analyses of PDAC cell lines treated with increasing concentrations of CHK1i for 24 h. (B) Clonogenic proliferation assay to monitor growth suppression of PDAC cell lines treated (8 days) with the indicated concentrations (nM) of CHK1i. (C) Anchorage-dependent growth of PDAC cell lines was evaluated by live cell counting following CHK1i treatment for 5 days. (D) The mean GI_50_ with SD of data shown in (C). (E and F) PDAC organoid growth was monitored by the CellTiter-Glo viability assay after treatment (10 days) with the indicated concentrations of CHK1i. (E) The median of three biological replicates for each treatment is shown, and a shift from blue to red indicates a reduction in growth. (F) Individual growth values are shown for each organoid line at each concentration of CHK1i. (G) The percentages of cells in the indicated phases of the cell cycle were determined using propidium iodide staining and flow cytometry following 24 h of treatment with the indicated CHK1i concentration (nM). (H) The percentage of cells undergoing apoptosis was evaluated in three PDAC lines with varying degrees of growth sensitivity to CHK1i (5 days). Apoptosis was monitored using fluorescence-activated cell sorting (FACS) analysis of Annexin V- and propidium iodide-labeled cells. Statistical significance was evaluated using one-way ANOVA and Dunnett’s multiple-comparisons test; **p < 0.01, ***p < 0.001, ****p < 0.0001. In (A)–(D), (G), and (H), all experiments were performed in biological triplicate, immunoblots are representative images, and graphs show mean and SD.

The necessity of CHK1 in PDAC was evaluated using the potent and selective CHK1 clinical candidate inhibitor prexasertib (CHK1i). We monitored inhibition of CHK1 by immunoblotting for increased CHK1 phosphorylation at S345 (pCHK1), a biochemical marker for CHK1 inhibition caused by loss of PP2A feedback inhibition (Leung-Pineda et al., 2006). We observed an increase in pCHK1 following CHK1i treatment, beginning at 2–4 nM (Figures 2A and S2A). We also monitored γH2AX as a functional marker of DNA DSBs. We observed a dose-dependent increase in CHK1 inhibition-induced γH2AX expression, supporting a CHK1i-mediated accumulation of DNA DSBs.

Inhibition of CHK1 suppressed growth in both colony formation (Figures 2B and S2B) and anchorage-dependent proliferation assays (Figures 2C, 2D, S2C, and S2D). The GI_50_ in sensitive cell lines (4–8 nM) was comparable with the half maximal inhibitory concentration (IC_50_) for CHK1i target inhibition, supporting on-target growth suppression (Figures 2C and 2D). The Pa16C cell line exhibited partial resistance (GI_50_ = 44.6 nM), whereas PANC-1 cells were resistant at CHK1i concentrations up to 200 nM (Figures 2D and S2C). CHK1i sensitivity was not associated with *TP53* mutation status, as all cell lines harbor *TP53* missense mutations (Figure S1A).

To address potential off-target activities of prexasertib, we evaluated a second CHK1 inhibitor, AZD7762, and we observed a similar pattern of sensitivity (Figure S2D). Levels of γH2AX correlated with CHK1i sensitivity (Figures 2A and S2A).

To address whether *KRAS* mutation status affected CHK1i sensitivity, we first evaluated sensitivity in two wild-type (WT) KRAS PDAC cell lines. BxPC-3 cells have an activated *BRAF* mutation and PATC-153 cells a *PIK3CA* activating mutation and consequently retain a partially activated RAS phenotype. BxPC-3 cells were highly sensitive (GI_50_ = 2.8 nM), whereas PATC-153 cells showed 13-fold reduced sensitivity (GI_50_ = 37.5 nM) (Figures S2E and S2F). Second, we compared CHK1i in hTERT-immortalized HPNE human pancreatic cells and a matched cell line stably expressing KRAS^G12D^ (Campbell et al., 2007). Although both lines were sensitive to CHK1i-induced growth suppression, the KRAS^G12D^-expressing cells exhibited a 2-fold lower GI_50_ than control cells (2.1 and 4 nM, respectively) and greater induction of γH2AX (Figures S2G and S2H). Together, these analyses suggest that mutant KRAS, potentially through hyperactivation of ERK MAPK signaling, can increase sensitivity to CHK1i.

PDAC patient-derived organoid cultures may more accurately model patient response to therapy (Boj et al., 2015; Tiriac et al., 2018). CHK1i showed variable efficacy in suppressing the proliferation in KRAS mutant PDAC organoid models, with GI_50_ values ranging from 4 to >32 nM (Figures 2E and 2F). Taken together, our findings support CHK1 as an effective therapeutic target for a subset of KRAS mutant PDAC.

We determined the basis for CHK1i treatment-mediated growth inhibition. Applying the CellTox Green cytotoxicity assay, we observed CHK1i treatment increased apoptosis (Figure S2I). We also applied flow cytometry analyses to monitor the impact of CHK1i on cell-cycle arrest and apoptosis (Figures 2G, 2H, S2J, and S2K). CHK1 loss/inhibition was previously reported to cause S-phase accumulation (Branigan et al., 2021; Di Franco et al., 2021; van Harten et al., 2019; Heidler et al., 2020; Yuan et al., 2018), consistent with the role of CHK1 in mediating transient inhibition of new origin firing in S phase (Moiseeva et al., 2019). We observed variable effects on both cell-cycle arrest and apoptosis that correlated with CHK1i sensitivity (GI_50_) to growth inhibition. The sensitive line Pa01C, CHK1i caused reduction of cells in G_1_ and G_2_/M phases, accumulation in S phase, and increased apoptosis (Figures 2G, 2H, S2J, and S2K). In contrast, the partially resistant Pa16C cells did not accumulate in S phase, nor did CHK1i induce pronounced apoptosis (Figures 2G, 2H, S2J, and S2K). Pa02C cell responses were intermediate, with only the highest doses of CHK1i initiating S-phase arrest and at 5 days showing dramatically more apoptosis than at 3 days (Figures 2G, 2H, S2J, and S2K). Thus, CHK1i treatment induced both cell-cycle arrest and apoptotic growth.

### CHK1 inhibition causes accumulation of DNA DSBs

**Figure 3.**
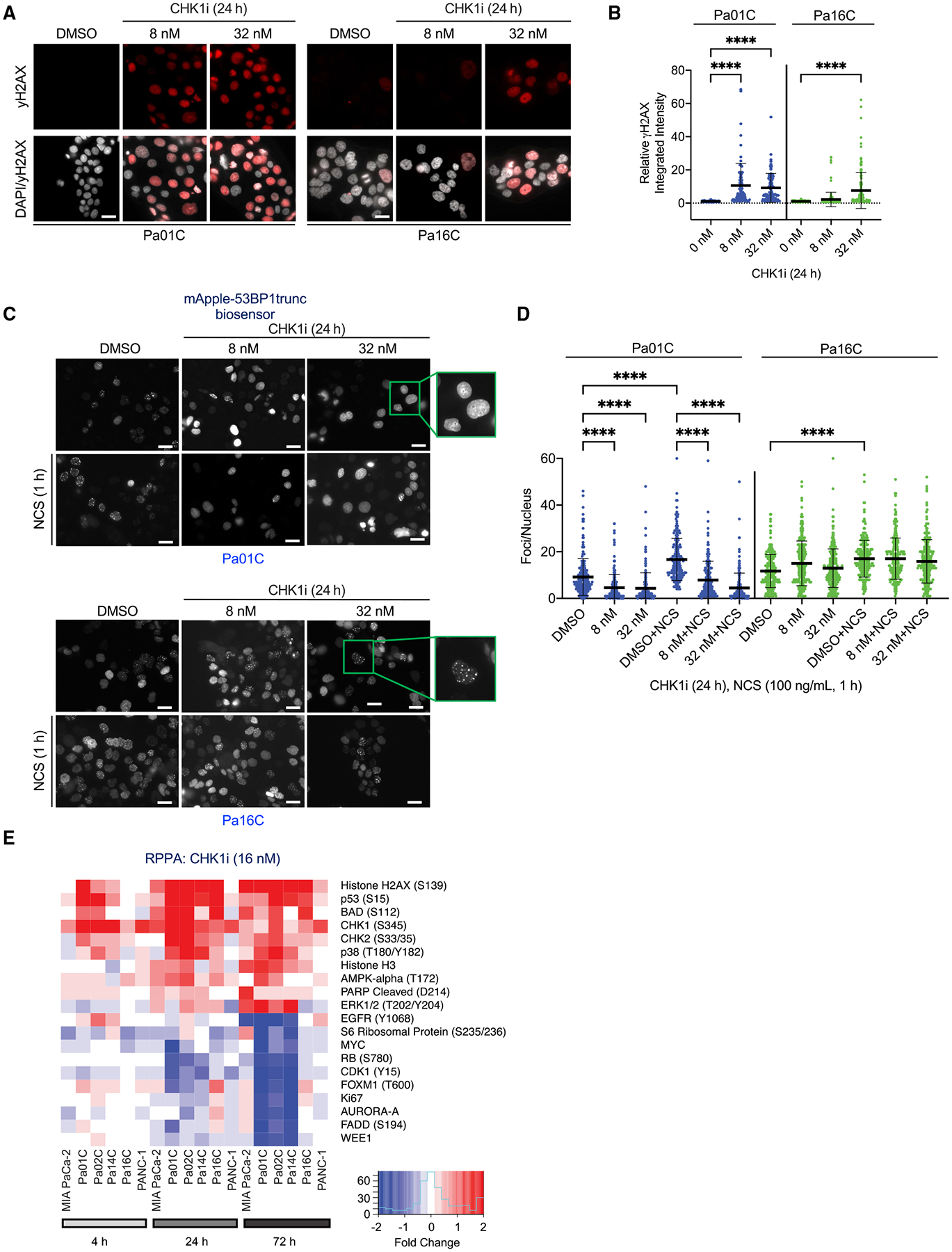
CHK1i promotes DNA damage and loss of 53BP1-mediated repair (A) Representative images of immunofluorescence to monitor γH2AX expression (red) and nuclei (white) in PDAC cells following CHK1i treatment (24 h) at the indicated concentrations (nM). Scale bar, 25 μm. (B) The relative integrated intensity of γH2AX per nucleus of the indicated cell lines treated with different doses of CHK1i. Each dot represents a nucleus, error bars represent the SD. Statistical significance was evaluated using one-way ANOVA with Dunnett’s multiple-comparisons test; ****p < 0.0001. (C) Representative images of Apple-tagged trunc53BP1 in PDAC cells following DMSO or CHK1i treatment for 24 h and/or the irradiation mimic neocarzinostatin (NCS) for 1 h at 100 ng/mL. Boxes show zoomed-in views with (Pa16C) or without (Pa01C) foci following CHK1i treatment. Scale bar, 25 μm. (D) The number of mApple-tagged trunc53BP1 foci per nucleus was evaluated. Statistical significance was evaluated using one-way ANOVA with Dunnett’s multiple-comparisons test; ****p < 0.0001. Each dot represents a nucleus and error bars the SD. (E) Heatmap of RPPA analyses to evaluate changes in the levels of phosphorylated (site[s] in parentheses) or total expression of the indicated proteins following CHK1i (15 nM) treatment for the indicated times in six PDAC cell lines. Shown are the median values from four biological replicates and highlights of the ten most up- and downregulated proteins.

Because CHK1 is a critical component of homologous recombination repair (HRR) (Sørensen et al., 2005), we investigated DNA damage following CHK1i. First, immunofluorescence analyses determined that the total intensity of γH2AX increased significantly in the sensitive Pa01C cell line at both 8 and 32 nM CHK1i, whereas in the more resistant Pa16C cells, the increase was significant only at 32 nM (Figures 3A and 3B). Additionally, the distribution of the increased γH2AX staining was predominantly pan-nuclear, an indicator of DNA damage-induced apoptosis (Ding et al., 2016) (Figures S3A and S3B).

We also applied a second assay for DNA damage to monitor the recruitment of p53-binding protein 1 (53BP1) to sites of DSBs, where it promotes non-homologous end joining (NHEJ) repair. 53BP1 is repressed during S and G_2_/M phases in order to inhibit error-prone NHEJ and instead promote accurate HRR of DSBs (Feng et al., 2015). To assess this, we stained for endogenous 53BP1 and used the mApple-53BP1trunc fluorescent biosensor (Yang et al., 2015). CHK1i decreased the number of 53BP1 foci in CHK1i-sensitive Pa01C but not resistant Pa16C cells (Figures 3C, 3D, S3C, and S3D). Additionally, the irradiation mimic neocarzinostatin (NCS), which causes SSBs and DSBs, increased the number of 53BP1 foci in both cell lines. However, this increase was blocked by CHK1i in sensitive Pa01C but not resistant Pa16C cells (Figures 3C, 3D, S3C, and S3D). Importantly, both the endogenous 53BP1 staining and the mApple-53BP1trunc biosensor showed similar results. As expected, 53BP1 foci (marker of NHEJ) colocalized with γH2AX foci (marker of DSBs) induced by NCS treatment (Figure S4A), whereas the formation of pan-nuclear γH2AX resulted in a loss of 53BP1 foci (Figure S4A). To determine the effects of CHK1i on HRR in Pa16C cells, RAD51 focus formation was evaluated. Treatment with NCS resulted in an increase in cells with three or more RAD51 foci. However, co-treatment with CHK1i blocked this increase, and CHK1i treatment alone also significantly decreased RAD51 foci containing cells below basal levels (Figures S4B and S4C). This is consistent with previous observations that CHK1 activity is required for HRR (Engelke et al., 2013; Morgan et al., 2010; Sørensen et al., 2005). Together, our results indicate that in CHK1i-sensitive PDAC cell lines, the loss of CHK1 function results in accumulation in S phase, loss of 53BP1-mediated NHEJ repair, increased formation of pan-nuclear γH2AX, and subsequent cell death. Furthermore, although CHK1i-resistant cells capable of escaping S-phase arrest can retain components of NHEJ repair, they lose RAD51-mediated HRR in the presence of CHK1i.

### CHK1 and ERK inhibition independently and in combination decrease MYC

To identify additional consequences of CHK1 inhibition, we applied reverse-phase protein array (RPPA) pathway activation mapping to monitor the phosphorylation/activation state and/or expression of cancer signaling network proteins (Baldelli et al., 2017). We treated PDAC lines with CHK1i and evaluated changes at 4, 24, and 72 h to identify both the immediate consequences of CHK1i as well as compensatory signaling activities (Figures 3E and S5). In agreement with our immunoblot analyses (Figures 2A and S2A), beginning at 4 h, CHK1i caused accumulation of DSBs, as indicated by increased γH2AX (Figures 3E, S5, and S6A–S6C). Consistent with CHK1i-induced apoptosis (Figures 2H, S2I, and S2K), we observed increases in phosphorylation and activation of the proapoptotic proteins BAD and BIM and in PARP cleavage.

We also identified a time-dependent reduction in MYC protein beginning at 4 h and maximal at 24 h of CHK1i treatment (Figures 3E, S5, and S6A–S6C). Immunoblot analyses observed that CHK1i (24 h) caused a dose-dependent decrease in MYC protein levels that correlated with CHK1i sensitivity (Figures 2A and S2A).

To determine a mechanistic basis for CHK1i-induced reduction in MYC, we determined a role for proteasome-dependent MYC protein degradation. ERK blocks MYC degradation by direct phosphorylation of MYC at S62 (Farrell and Sears, 2014; Vaseva et al., 2018), and conversely ERKi promotes loss of MYC by enhancing its degradation. However, whereas CHK1i treatment increased pS62 in CHK1i-resistant Pa16C cells, there was little change in CHK1i-sensitive Pa01C cells (Figures S6D and S6E). Furthermore, although treatment with the proteasome inhibitor MG132 increased the basal level of MYC, we found that CHK1i caused comparable reductions in MYC protein in the presence of MG132 (Figures S6D and S6E). Thus, CHK1i-mediated MYC loss does not involve KRAS-ERK signaling or an E3 ligase-dependent degradation mechanism.

In contrast, qRT-PCR analyses revealed that *MYC* transcription was reduced upon CHK1i treatment (Figure S6F). Furthermore, in the CHK1i-resistant Pa16C cells, we observed an accumulation of MYC transcript in the presence of cycloheximide (CHX) that was not observed in the sensitive Pa01C cells. Thus, CHK1i-mediated loss of MYC was primarily at the level of gene transcription.

### Concurrent inhibition of CHK1 and ERK causes enhanced growth suppression and apoptosis

RPPA analyses also revealed that CHK1i induced increases in pERK (Figures S5, S6A, and S6B). Although pERK was reduced initially following short-term CHK1i treatment (4 h), it rebounded and was increased at 24 and 72 h. Immunoblot analyses verified dose-dependent induction of pERK (Figure 2A).

**Figure 4.**
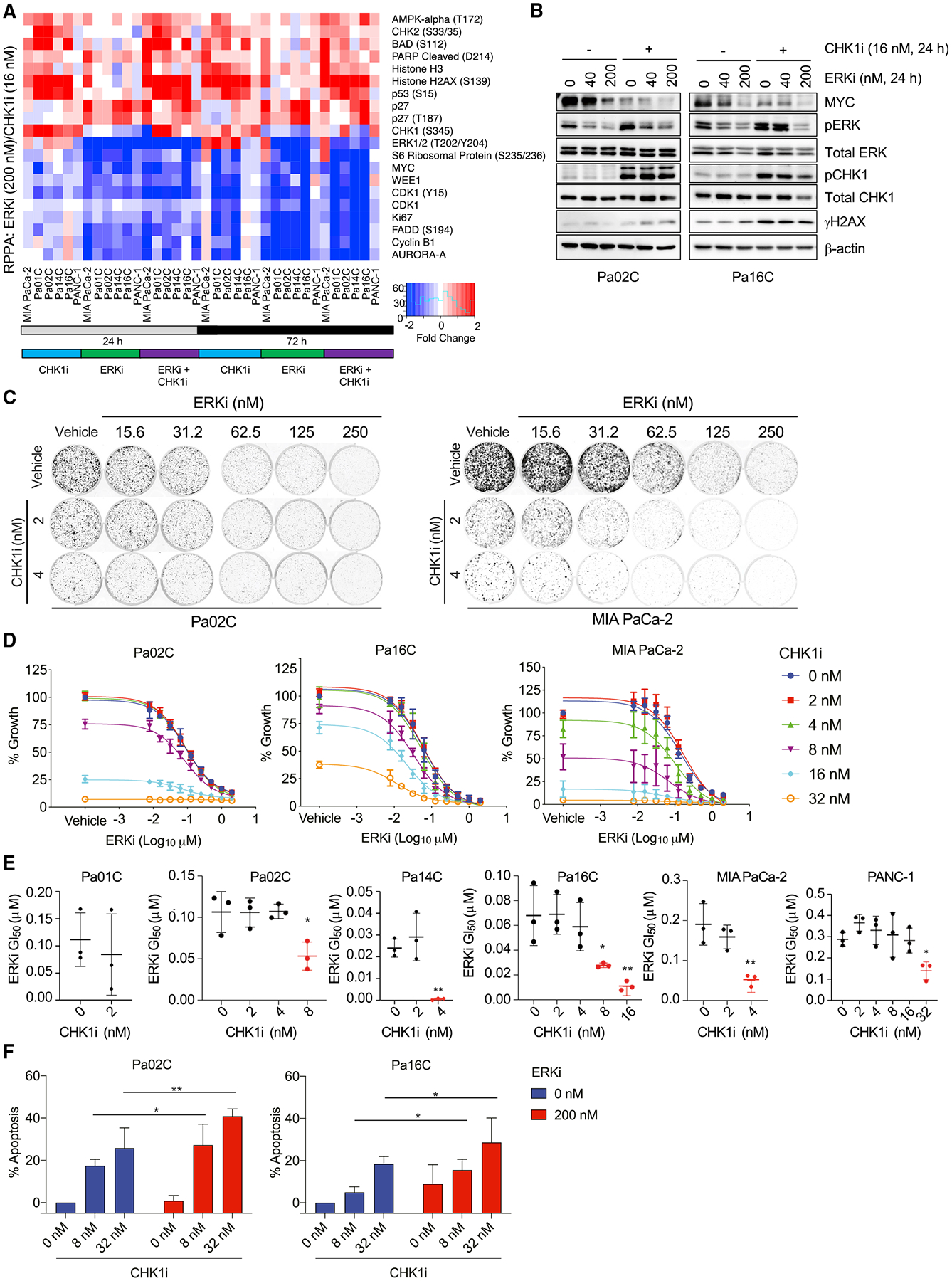
Concurrent CHK1i treatment enhances ERKi-mediated growth suppression and apoptosis (A) RPPA analyses of PDAC cell lines following 24 or 72 h treatment with CHK1i (15 nM) and/or ERKi (200 nM). The heatmap depicts the median values from four biological replicates and the ten most up- and downregulated protein changes on the basis of mean values of all cell lines evaluated. (B) Immunoblot analyses to monitor the indicated phosphorylated/total protein levels in cells treated (24 h) with the indicated concentrations of CHK1 and/or ERKi. (C) Clonogenic growth assay of PDAC cell lines treated for 8 days with the indicated inhibitor concentrations. Cells were visualized using staining with crystal violet. (D) Growth of PDAC cell lines was evaluated using live cell counting following CHK1i and/or ERKi treatment for 5 days. (E) The mean ERKi GI_50_ was determined following treatment with different concentrations of CHK1i. One-way ANOVA with Dunnett’s multiple-comparisons test was used to determine significance; *p < 0.05, **p < 0.01. (F) Percentage of cells undergoing apoptosis induced by treatment with CHK1i and/or ERKi was determined using FACS analysis of Annexin V- and propidium iodide-labeled cells. Significance was determined using two-way ANOVA and Tukey’s multiple-comparisons test; *p < 0.05, **p < 0.01. In (B)–(F), all experiments were performed in biological triplicate, for immunoblots a representative image is shown, and graphs depict mean and SD.

CHK1i-induced compensatory ERK activation is consistent with our identification of *CHEK1* loss as enhancing ERKi-mediated growth suppression (Figures 1B and 1C). RPPA analyses showed that whereas CHK1i alone increased pERK (Figure 4A), when combined with ERKi, pERK was suppressed to the same level as observed upon ERKi treatment alone (Figures 4A and 4B). The combination induced markers for both G_1_ cell-cycle arrest and apoptosis (Figure 4A).

Colony growth and proliferation assays were performed to evaluate the consequences of concurrent CHK1i and ERKi treatment on a panel of ERKi sensitive (Pa02C, Pa14C, MIA PaCa-2) or resistant (Pa01C, Pa16C, PANC-1) PDAC cell lines (Hayes et al., 2016). CHK1i sensitized four of the six cell lines to ERKi treatment (Figures 4C, 4D, and S7A–S7C), and addition of CHK1i resulted in a significant decrease in ERKi GI_50_ (Figure 4E). Similar results were observed upon concurrent ERK and CHK1 inhibition using the ERKi (SCH772984) and a second CHK1 inhibitor, AZD7762 (Figure S7D). The outliers were Pa01C cells, for which growth was nearly completely blocked by the lowest dose of CHK1i alone, and PANC-1 cells, for which even the highest dose of CHK1i or ERKi had little effect (Figures 4E, S7A, and S7B).

Activation of ERK in response to DNA damage has been shown to be dependent on AKT (Khalil et al., 2011), and our RPPA analyses showed that CHK1i delayed the onset of AKT activation (Figures S5 and S6A). Treatment with the AKT inhibitor ipatasertib (AKTi) blocked CHK1i-induced pERK in Pa02C but not Pa01C cells (Figure S7E). Treatment with the MEK inhibitor trametinib (MEKi) also blocked CHK1i induction of pERK in all cell lines, indicating a mechanism upstream of ERK. Finally, whereas AKTi treatment did not block CHK1i-induced pERK induction in Pa01C cells, we observed decreases in the ERK negative regulator DUSP6 (Figure S7E), suggesting an additional mechanism to increase pERK.

Combination CHK1i and ERKi treatment caused strong MYC suppression (Figure 4B). To address a role for MYC in CHK1i and/or ERKi growth suppression, we established PDAC cells stably overexpressing WT MYC (Figure S7F). As described previously (Vaseva et al., 2018), ectopic MYC expression reduced ERKi growth inhibition (Figure S7G). However, although the degree of MYC loss correlated strongly with CHK1i sensitivity (Figures 2A and S2A), neither CHK1i nor combined CHK1i and ERKi growth suppression was reversed by MYC overexpression (Figures S7H and S7I). Thus, the enhanced loss of MYC by concurrent CHK1i treatment was a key driver of ERK-dependent but not CHK1-dependent growth.

Finally, ERKi in combination with the clinical candidate ATR inhibitor AZD6738/ceralasertib (ATRi) showed similar results as ERKi+CHK1i (Figure S7J). ERK and CHK1i/ATRi predominantly caused additivity, but some synergy was observed (Table S4). Consistent with the RPPA results, we also observed a significant increase in apoptosis upon combined ERK and CHK1 inhibition (Figure 4F). We conclude that combined inhibition of ERK and CHK1 kinase activities results in a more potent blockade of cell growth as well as increased apoptosis.

### CHK1 expression is downregulated by KRAS-ERK inhibition-mediated G1 arrest

**Figure 5.**
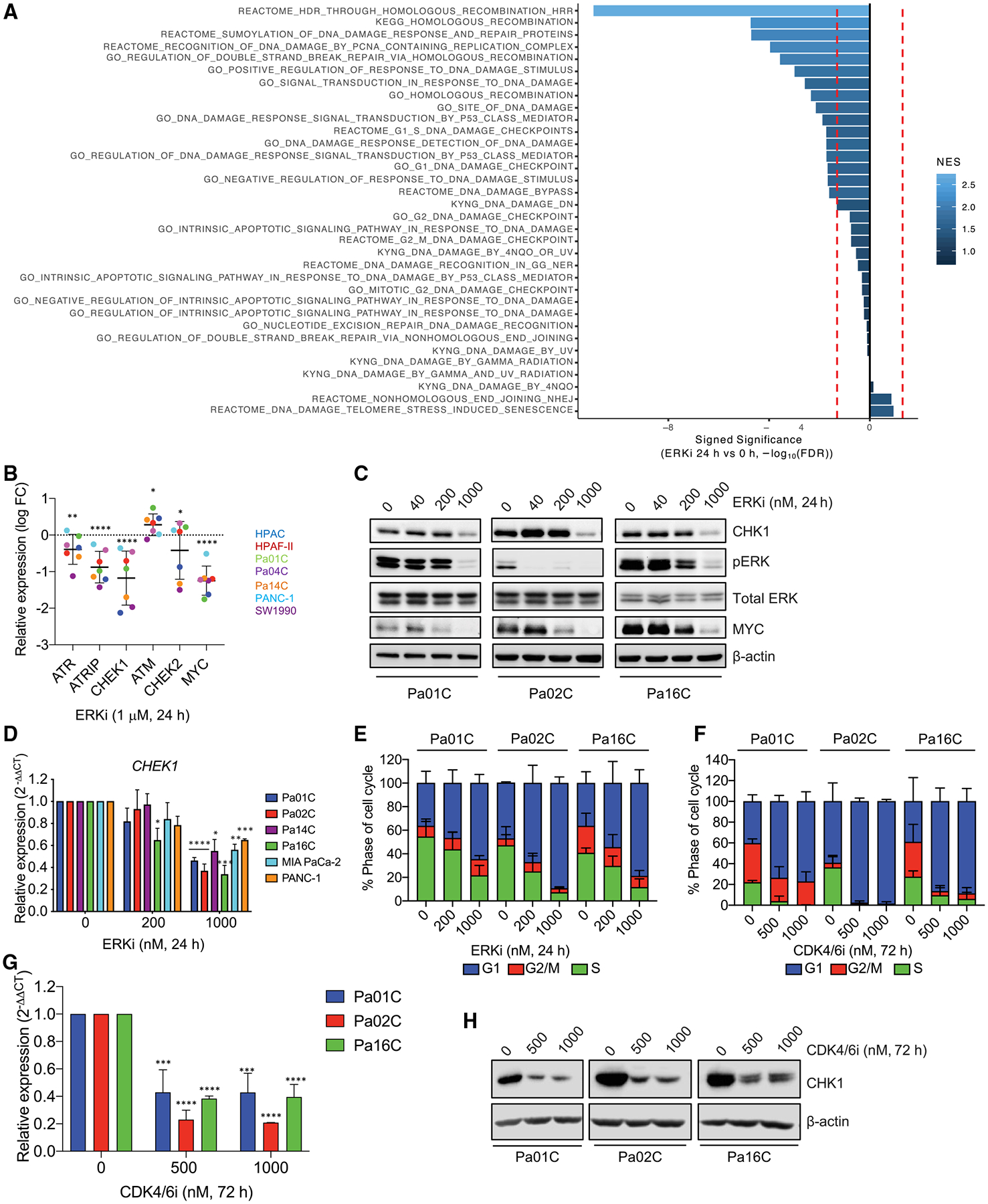
ERK inhibition decreases CHEK1 gene and CHK1 protein expression by causing G1 cell-cycle arrest (A) Normalized gene set enrichment statistics were calculated using ranked log fold change (FC) values with our previous RNA-seq data of HPAC, HPAF-II, Pa01C, Pa04C, Pa14C, PANC-1, and SW1990 cell lines treated with ERKi (1 μM, 24 h) compared with baseline (0 h) (PRJEB25806) (Bryant et al., 2019). Shown is enrichment for 34 DNA damage repair gene sets from the Molecular Signatures Database (MSigDB) in ERKi-treated cells. Negative enrichment indicates genes downregulated upon the addition of ERKi. Gene sets used in analysis are provided in Table S2. (B) Relative mRNA expression of the indicated DDR genes from RNA-seq analyses of the indicated PDAC cell lines treated with ERKi (1 μM, 24 h). All values were standardized to 0 h to determine the relative expression change. The mean and SD were determined, with each dot representing a different cell line. *MYC* expression change was evaluated as a positive control for a previously validated ERK-regulated gene (Vaseva et al., 2018). Statistical significance was determined via dispersion corrected, moderated t tests as implemented in limma;*p < 0.05, **p < 0.01, ***p < 0.001, ****p < 0.0001. (C) PDAC cells were treated with the indicated concentrations of ERKi (24 h) and evaluated using immunoblot analyses for the indicated proteins. (D) Relative expression of *CHEK1* in the indicated PDAC cell lines after treatment (24 h) with the indicated ERKi concentrations analyzed via qRT-PCR. Significance was determined by a two-way ANOVA and Dunnett’s multiple comparison test; *p < 0.05, **p < 0.01, ***p < 0.001, ****p < 0.0001. (E) Flow cytometry analyses to determine the percentage of cells in specific phases of the cell cycle, in PDAC cell lines treated (24 h) with the indicated concentrations of ERKi. (F) Flow cytometry analyses to determine the percentage of cells in specific phases of the cell cycle, in PDAC cell lines treated (24 h) with the indicated concentrations of palbociclib (CDK4/6i). (G) qRT-PCR analyses to determine *CHEK1* transcript levels following 24 h palbociclib (CDK4/6i) treatment of PDAC cell lines at the indicated concentrations. Significance was determined by a two-way ANOVA and Dunnett’s multiple comparison test; ***p < 0.001, ****p < 0.0001. (H) Immunoblot analyses to determine CHK1 protein levels following 72 h palbociclib (CDK4/6i) treatment of PDAC cell lines at the indicated concentrations. In (C)–(H), all experiments were performed in biological triplicate, for immunoblots a representative image is shown, and graphs depict mean and SD.

CHK1 is required for HRR (Sørensen et al., 2005), and we observed CHK1i-induced ERK activation as a compensatory response to CHK1 inhibition (Figures 2A and 3E). Therefore, we speculated that ERK may regulate HRR. To address this possibility, we evaluated RNA-seq data from seven KRAS mutant PDAC cell lines following 24 h of ERKi treatment (Bryant et al., 2019) and observed downregulation of genes involved in HRR (Figure 5A). Specifically, transcripts of *CHEK1* as well as *ATR* and *ATRIP* (essential binding partner of ATR) were decreased following ERK inhibition, whereas *ATM* levels were elevated and changes in *CHEK2* were variable (Figures 5B and S8A). RPPA analyses showed that pCHK1 and pATR, but not pATM or pCHK2, were decreased following ERK inhibition (Figure S8B).

To validate the RNA-seq expression data, we performed immunoblot analyses and determined that *CHEK1*/CHK1 decreased at the RNA and protein levels following *KRAS* depletion or ERKi treatment (Figures 5C, 5D, and S8E–S8G). At 4 h of ERKi, we observed a decrease in phosphorylated but not total CHK1 (Figure S8D), indicating that loss of CHK1 expression occurs only after prolonged KRAS-ERK suppression. ERKi-induced reduction in CHK1 levels provides another basis for the enhanced potency of ERKi observed upon concurrent CHK1i treatment.

As KRAS or ERK inhibition causes G_1_ arrest in KRAS mutant PDAC cell lines (Hayes et al., 2016; Lee et al., 2019) (Figures 5E and S8H), we speculated that CHK1 loss may be indirect and not due specifically to loss of ERK signaling. To address this possibility, we used the CDK4/6 inhibitor palbociclib to arrest PDAC cells in G_1_, independent of ERK signaling, and also observed loss of *CHEK1* RNA and CHK1 protein (Figures 5F–5H and S8I). Thus, the reduction in CHK1 expression is likely a consequence of the resulting G_1_ arrest rather than a consequence of direct loss of KRAS-ERK signaling.

### Loss of RIF1 increases sensitivity to CHK1i

**Figure 6.**
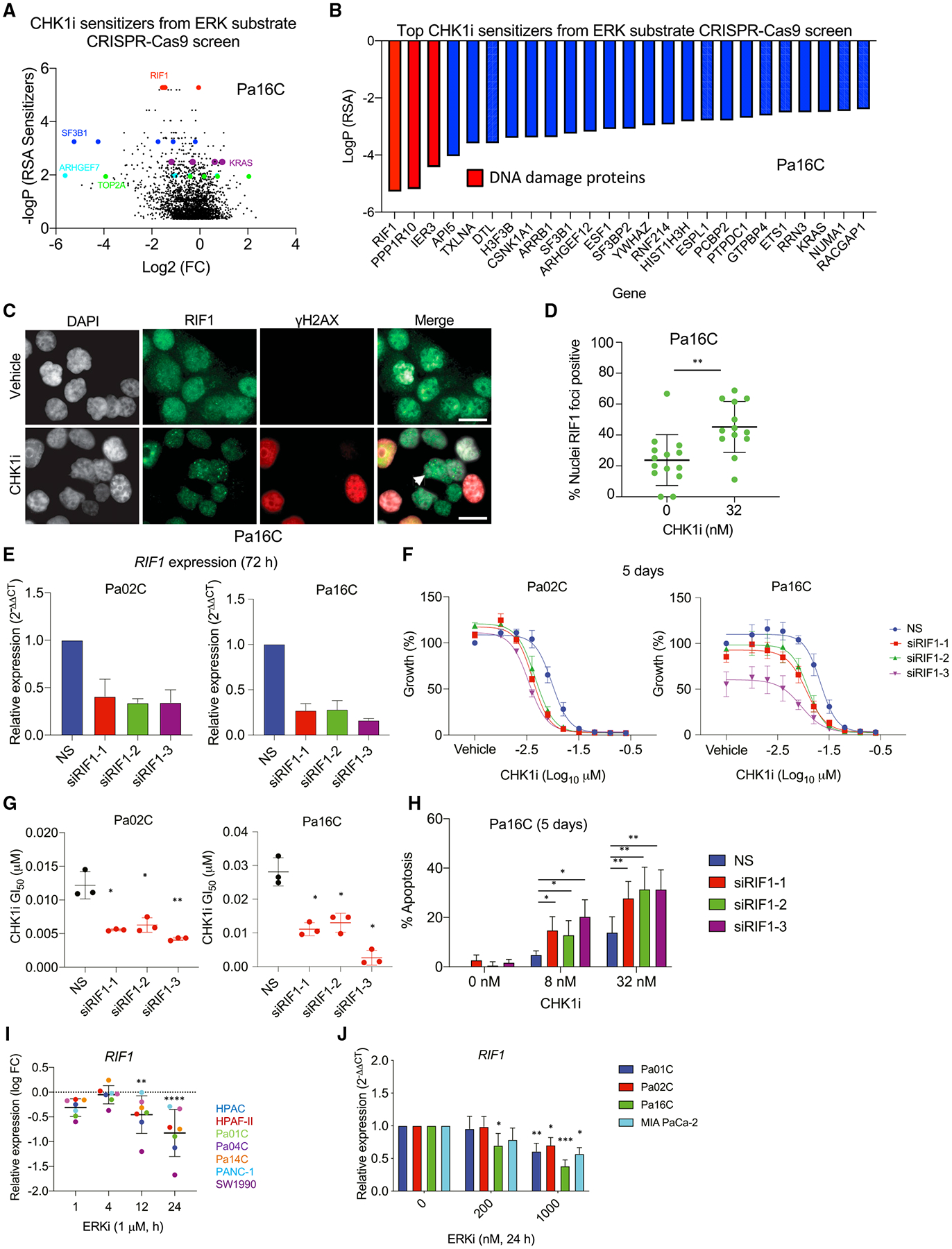
Loss of RIF1 increases sensitivity to CHK1i (A) Shown is a volcano plot comparing the log_2_ fold change (FC) versus the −logP (RSA sensitizer) of data from the CRISPR-Cas9 ERK substrate library screen to identify genes that modulate sensitivity to treatment with CHK1i at GI_25_ (15 nM, 4 weeks) or with DMSO vehicle control. (B) Top 25 genes identified in the screen in (A) were ranked by logP following RSA analysis, relative to vehicle-treated cells. (C) Representative immunofluorescence images of RIF1 (green) and γH2AX (red) expression following CHK1i or vehicle treatment. DAPI staining was done to visualize nuclei (white). Arrowhead indicates a RIF1 foci-positive nucleus; scale bar, 25 μm. (D) Quantification of the percentage of RIF-positive nuclei. Significance was evaluated using an unpaired t test; **p < 0.01. (E) Relative *RIF1* expression was determined using qRT-PCR to quantify knockdown after treatment (72 h) with three distinct siRNAs targeting *RIF1* or NS control in Pa02C and Pa16C PDAC cell lines further characterized in (F)–(H). (F) Growth was evaluated using live cell counting following RIF1 knockdown and CHK1i treatment (5 days). Cells were reverse-transfected with NS or three different siRNAs targeting *RIF1* and treated with CHK1i starting 12 h later. (G) Graph showing the GI_50_ for CHK1i in NS versus RIF1 knockdown cells as in (F). Significance was determined by one-way ANOVA with Dunnett’s multiple-comparisons test; *p < 0.05, **p < 0.01. (H) *RIF1* was depleted in Pa16C cells via siRNA for 12 h, and then cells were treated with CHK1i for an additional 5 days. Apoptosis was monitored using FACS analysis of propidium iodide and fluorescein isothiocyanate (FITC)-Annexin-stained cells. Significance was determined using two-way ANOVA and Tukey’ s multiple-comparisons test; *p < 0.05, **p < 0.01. (I) Cells were treated with 1 μM ERKi for the indicated times. RNA was collected and analyzed using RNA-seq. Statistical significance was determined using dispersion corrected, moderated t tests as implemented in limma; **p < 0.01, ***p < 0.001. Each dot represents a cell line. (J) Cells were treated with the indicated concentrations of ERKi for 24 h, then RNA was collected and evaluated using qRT-PCR. Significance was determined using one-way ANOVA and Dunnett’s multiple-comparisons test, where each treatment was compared with DMSO treatment; *p < 0.05, **p < 0.01, ***p < 0.001. In (C)–(H) and (J), all experiments were performed in biological triplicate. and graphs depict mean and SD.

To determine if the potent growth blockade resulting from concurrent CHK1i and ERKi treatment can be ascribed to any components downstream of ERK, we constructed a 1,223-gene CRISPR-Cas9 library targeting known and putative ERK substrates (Ünal et al., 2017). This library was used to perform a loss-of-function screen in Pa16C cells treated with a sublethal dose of CHK1i (8 nM). CHK1i sensitizers were identified (Figures 6A, 6B, and S9A), and as expected, *KRAS* (included in the library as a control) was among them. The top three hits were genes involved in DDR, *RIF1*, *PPP1R10*, and *IER3*. IER3 is a known ERK downstream effector (Cano et al., 2014; Hamidi et al., 2012) and has been shown to help maintain CHK1 activation (Pawlikowska et al., 2010). *PPP1R10* has also been linked previously to CHK1i activity (Landsverk et al., 2010). As little is known about *RIF1* in PDAC, we evaluated the relationship between RIF1 and CHK1.

In response to DNA damage, RIF1 interacts with 53BP1 to promote, in an ATM-dependent manner, the formation of foci that enable NHEJ-mediated repair of DSBs (Escribano-Díaz et al., 2013; Silverman et al., 2004). CHK1i treatment did not induce RIF1 foci formation in the CHK1i-sensitive cell line Pa01C (Figures S9B and S9C). This was likely due to CHK1i-induced S phase accumulation (Figure 2G) and therefore inhibition of NHEJ-mediated repair, resulting in a decrease in 53BP1 foci (Figures 3C, 3D, S3C, and S3D). Conversely, CHK1i treatment of resistant Pa16C cells resulted in a significant increase in RIF1 recruitment (Figures 6C and 6D) as well as no depletion in the number of 53BP1 foci (Figures 3C, 3D, S3C, and S3D). We also determined that neither ERKi treatment or *RIF1* suppression alone affected formation of 53BP1 foci, but combining either with CHK1i reduced the numbers of 53BP1 foci in Pa16C cells (Figures S9D–S9G).

Furthermore, siRNA depletion of *RIF1* sensitized cells to CHK1i-mediated growth suppression and resulted in a significant decrease in the GI_50_ for CHK1i, as well as an increase in apoptosis (Figures 6D–6G). The increased sensitivity to RIF1 loss in the presence of CHK1i is also bolstered by the blockade of RAD51 foci formation via CHK1i (Figures S4B and S4C). To date, no studies have addressed how ERK may affect RIF1 expression or function. However, our analyses demonstrated that ERKi decreased *RIF1* transcription (Figures 6I and 6J). These results validate our identification of RIF1 in the ERK substrate loss-of-function screen as a sensitizer to CHK1 inhibitor. Furthermore, we found that RIF1 depletion phenocopied ERKi in all endpoints assessed.

### CHK1 inhibition induces autophagy

**Figure 7.**
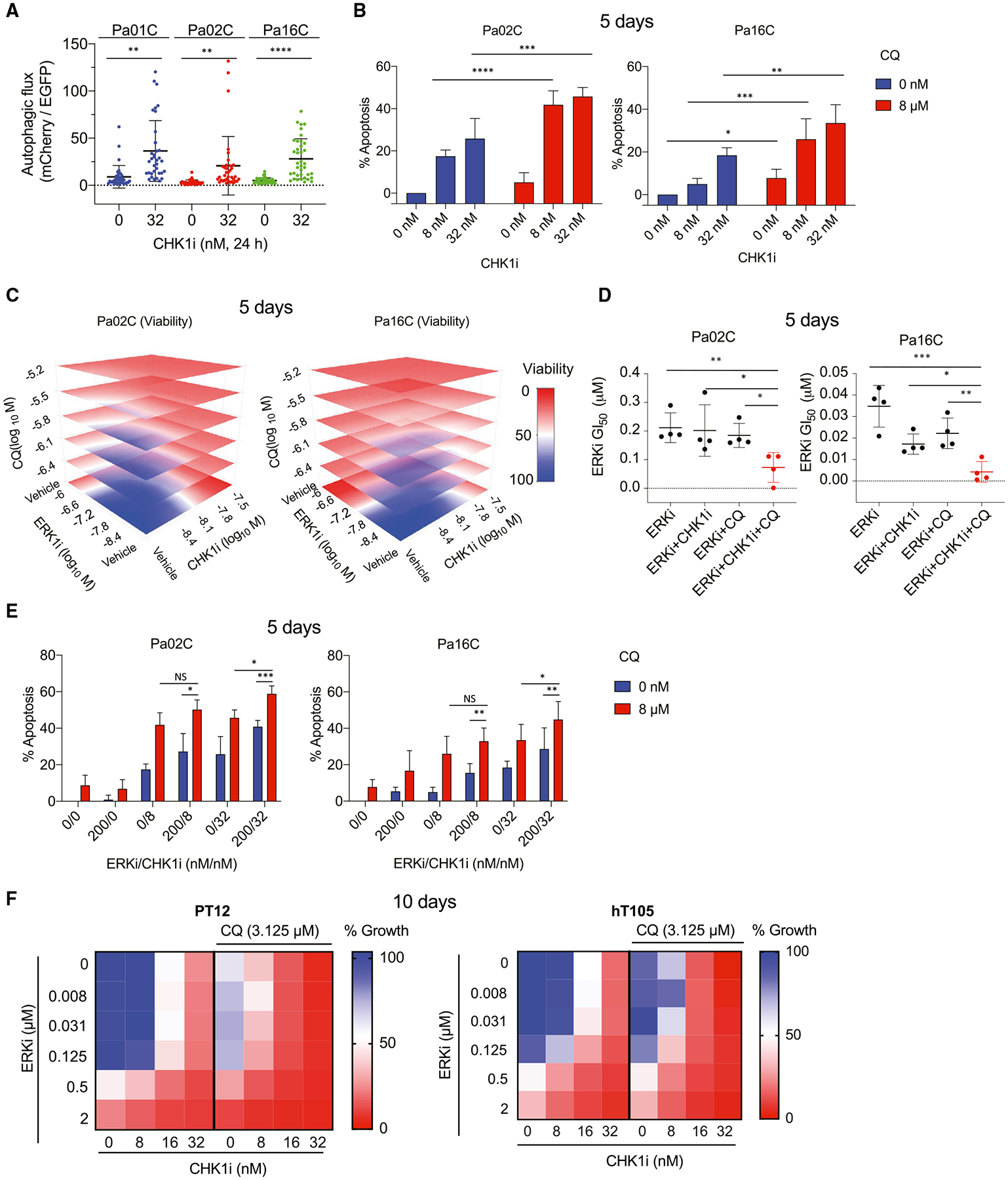
CHK1 inhibition induces autophagy (A) Cell lines stably expressing the autophagic flux biosensor mCherry-EGFP-LC3B were imaged following treatment with CHK1i (32 nM, 24 h) or vehicle control. Significance was evaluated using an unpaired t test; **p < 0.01, ****p <0.0001. Representative images are shown in Figure S10A. (B) Percentage of cells in apoptosis induced by CHK1i and/or chloroquine (CQ) alone or in combination (5 days) was determined via FITC-Annexin V staining and flow cytometry. Significance was determined using two-way ANOVA and Tukey’s multiple-comparisons test; *p < 0.05, **p < 0.01, ***p < 0.001. (C) Cells were treated for 5 days with the indicated concentrations of ERKi, CHK1i, and/or CQ alone or in combination. Cell growth was evaluated using live cell counting. For the kill effect, a shift from blue to red indicates a decrease in viability. (D) Graph showing alterations in the GI_50_ of ERKi following treatment combinations as shown in (C). GI_50_ shifts are shown for Pa02C and Pa16C cells treated with CHK1i at 4 and 8 nM, respectively, and 1.56 μM of CQ. Significance was determined as in (B). (E) Cells were treated simultaneously with CHK1i (8 or 32 nM) and ERKi (200 nM), with or without CQ (8 μM) for 5 days. Cells were collected, and apoptosis was determined using FACS analysis of Annexin V- and propidium iodide-labeled cells. Significance was determined as in (B). (F) The same triple combinations as in (C)–(E) were evaluated in patient-derived PDAC organoids. Organoids were treated for 10 days with the indicated concentrations of ERKi and CHK1i, with or without CQ (3.125 μM). The median of three biological replicates for each treatment is shown, and a shift from blue to red indicates reduction in organoid viability as assessed using CellTiter Glo. In (A)–(E) all experiments were performed in biological triplicate (A–D) or quadruplicate (E), and graphs represent the mean and SD.

We found that CHK1i treatment caused an upregulation of phosphorylated and activated AMPK⍺ (T172) (Figures 2A, 3E, and 4A). One well-characterized activity of AMPK is phosphorylation and inactivation of mTORC1 to stimulate autophagy. We therefore reasoned that, in PDAC, CHK1 may be involved in regulation of autophagy, a metabolic process of nutrient acquisition. To determine if CHK1i stimulated autophagy, we used the dual fluorescent probe mCherry-EGFP-LC3B to monitor autophagic flux and found a significant increase in autophagic flux (Figures 7A and S10A).

This finding is similar to our recent determination that ERK inhibition increased autophagy (Bryant et al., 2019) and consequently increased sensitivity to the autophagy inhibitor chloroquine (CQ). We therefore first determined if concurrent treatment with CQ would enhance CHK1i growth inhibitory activity. Concurrent treatment with CHK1i and CQ dose-dependently suppressed growth and increased apoptotic death in PDAC cell lines (Figures 7B, S10B, and S10C). ATRi and CQ showed very similar results (Figure S10D), and in both cases additivity was predominantly observed with some synergy (Table S4).

We showed previously that CQ sensitizes cells to ERKi-induced growth suppression but does not result in substantial apoptosis (Bryant et al., 2019). In this study, we found that CHK1i also sensitized cells to ERKi and that CQ sensitized cells to CHK1i (Figures S10B and S10C). Furthermore, we found that combining CHK1i with either ERKi (Figure 4F) or CQ (Figure 7B) promoted apoptosis. We therefore determined if a triple inhibitor combination of CHK1i, ERKi, and CQ would exhibit further potency. The triple combination caused synergistic growth suppression (Figures 7C and S10E) and caused a significant decrease in ERKi GI_50_ compared with the double combinations of ERKi and CQ or ERKi and CHK1i (Figures 7D and S10F). Additionally, the triple combination resulted in significantly higher cell death than single agent or any dual inhibitor combination (Figure 7E). However, only minimal cell death was observed in NIH 3T3 cells compared with PDAC lines with the triple combinations (Figure S10G). Extending these analyses to patient-derived PDAC organoid cultures, we found that the triple combination also potently suppressed growth of these organoids (Figures 7F and S10H). In both cell lines and organoids, a combination of additivity and synergy was observed (Table S4). We conclude that, like KRAS-ERK inhibition, CHK1 inhibition upregulates autophagy dependence, creating an exploitable therapeutic vulnerability in PDAC.

## DISCUSSION

The RAF-MEK-ERK signaling network is a driver of KRAS-dependent cancer growth, and ERK inhibition is an effective therapeutic approach for KRAS mutant PDAC (Hayes et al., 2016). However, treatment-induced cancer cell acquired resistance and normal cell toxicity limit the effectiveness of ERK inhibitors. To overcome these limitations, we applied unbiased functional screens to identify combinations that enhance ERKi efficacy. We determined that loss of CHK1 function enhanced ERKi-mediated growth suppression of KRAS mutant PDAC cell lines and organoids. We identified multiple mechanisms that contribute to the combination, one of which was the dual blockade of both RIF1-mediated NHEJ repair and RAD51-mediated HRR. Additionally, we found that CHK1i activated AMPK and stimulated autophagy, and that concurrent inhibition of CHK1 and autophagy also caused growth suppression. Finally, the triple combination of inhibitors of CHK1, ERK, and autophagy caused even further growth suppression. In summary, while inhibition of CHK1 has been considered for use with therapies that cause DNA damage, our findings also support the use of CHK1 inhibitors together with inhibitors of KRAS effector signaling and autophagy.

Our identification of CHK1i as a strategy to enhance the anti-tumor activity of ERK inhibitor treatment contrasts with a previous study of non-PDAC cancers, in which ERK MAPK signaling was associated with CHK1i sensitivity and MEK inhibition drove CHK1i resistance (Lee et al., 2017). However, our findings are consistent with those of studies of multiple myeloma, in which concurrent inhibition of MEK and CHK1 caused synergistic growth suppression *in vitro* and *in vivo* (Dai et al., 2008; Pei et al., 2011).

One mechanistic basis for the efficacy of the ERKi and CHK1i combination in PDAC involves convergence on CHK1 function. We found that ERKi suppressed *CHEK1* gene transcription, complementing inhibition of CHK1 catalytic function. This mechanism corroborates the previous observation that MEK1/2 inhibition radiosensitized PDAC cells and led to the suppression of both NHEJ and HRR proteins (Estrada-Bernal et al., 2015). A second mechanistic basis for this synergy may involve ERKi-mediated inhibition of glycolysis. We and others demonstrated previously that ERK-MEK inhibition resulted in downregulation of glycolysis (Bryant et al., 2019; Ying et al., 2012). Dual inhibition of CHK1 and the GLUT1 glucose transporter is synergistically cytotoxic in KRAS mutant cancer cells (Erber et al., 2019). Thus, both inhibition of ERK and inhibition of GLUT1 result in a glycolytic phenotype, and this may contribute to the synergy of concurrent ERK and CHK1 inhibition. Relatedly, our observations of decreased levels of CHK1 following ERKi treatment may also be due to the dampening of glycolysis. Glucose deprivation alone results in increased degradation of CHK1 through the ubiquitin-proteasome pathway (Ma et al., 2019).

A third potential mechanistic basis for the synergistic activity of concurrent CHK1 and ERK inhibition involves the ERK activation observed following CHK1i alone. Similar to previous studies (Dai et al., 2008; Dent et al., 2011; Lee et al., 2017), we identified paradoxical activation of ERK as a potential compensatory response to overcome CHK1i-associated growth suppression. We showed recently that ERKi caused loss of MYC protein, primarily through stimulation of protein degradation (Vaseva et al., 2018). Loss of MYC following CHK1i has been described in RAS WT cancers (Ferrao et al., 2012; Krüger et al., 2018; Ravi et al., 2016). However, as CHK1i elevated ERK activity, which is a driver of MYC protein stability, loss of MYC was not expected in KRAS mutant PDAC. Given our finding that CHK1i reduced MYC expression through decreased transcription, we suggest that combining ERK and CHK1 inhibition causes distinct but complementary mechanisms to drive MYC loss.

CHK1i-mediated DNA damage may induce compensatory ERK activation to dampen induction of apoptosis through recruitment of RIF1 to DSBs and for NHEJ repair (Escribano-Díaz et al., 2013; Silverman et al., 2004). However, as this repair mechanism is blocked in S-phase arrested cells, CHK1i-sensitive cells are unable to combat apoptosis. When inhibition of CHK1 causes cells to accumulate in S phase, foci formation of RIF1–53BP1 is antagonized by BRCA1, and cells switch from relying on NHEJ-mediated repair to relying on HRR-mediated repair (Chapman et al., 2013). However, CHK1 activity is required for efficient HRR activity (Brill et al., 2017; Sørensen et al., 2005), as demonstrated by the block in RAD51 foci formation upon CHK1i treatment. Consequently, only cell lines in which inhibition of CHK1 does not induce S-phase accumulation are able to engage in NHEJ-mediated repair and escape apoptosis. Also, inhibition of ATR or CHK1 can result in increased phosphorylation of RIF1 by CDK1, promoting dissociation of RIF1 from PP1, allowing increased replication fork origin firing (Moiseeva et al., 2019). Loss of RIF1-PP1 also results in replication fork exposure, leading to increases in genome instability (Mukherjee et al., 2019). RIF1-PP1 is also essential for DNA bridging, which is crucial for cells experiencing oncogene-induced replicative stress (Bhowmick et al., 2019). Therefore, in addition to its essentiality for NHEJ-mediated repair, RIF1 is also critical for replication fork protection. We found that loss of RIF1 expression, whether from ERK inhibition or genetic depletion, sensitized otherwise CHK1i-resistant PDAC cell lines. In conclusion, we propose that, when CHK1 inhibition is not sufficient to induce S-phase arrest, PDAC cells then rely on RIF1 for NHEJ and/or replication fork protection to escape from CHK1i-induced apoptosis.

We also identified activation of AMPK and induction of autophagy as responses to CHK1i treatment. How CHK1i leads to AMPK activation is not known. Nevertheless, similar to our recent finding with ERK inhibition (Bryant et al., 2019), we observed that CHK1 inhibition increased autophagic flux and sensitized KRAS mutant PDAC cells to inhibition of autophagy. Our results support and extend the observation that lysosomal inhibition with the autophagy inhibitor CQ sensitized PDAC cells to inhibitors of the replication stress response (Elliott et al., 2019). Furthermore, they build on previous observations that autophagy-deficient mouse fibroblasts decreased the use of HRR and became dependent on NHEJ repair (Liu et al., 2015). The reduction of HRR was shown to be the result of decreased CHK1 levels because of increased proteasomal activity. Thus, it is likely that CQ treatment decreases HRR via reducing CHK1 expression, thereby sensitizing PDAC cells to CHK1i and enhancing CHK1i-mediated growth suppression and apoptosis.

We recently showed that ERKi stimulated autophagy as a compensatory response to the suppression of other metabolic processes (e.g., glycolysis, mitochondrial function). Stimulation of autophagy by CHK1i in PDAC may be caused by a distinct mechanism. CHK1i treatment causes formation of micronuclei in other cancer types (Kubara et al., 2012; Lewis and Golsteyn, 2016; Mani et al., 2019). Although a detailed mechanism for the cellular removal of micronuclei is unknown, it likely involves autophagy upregulation to clear CHK1 inhibition-induced accumulation of DNA damage (Rello-Varona et al., 2012). Furthermore, blockage of chaperone-mediated autophagy renders cells unable to initiate DNA repair because of hyperphosphorylation of the MRE11-RAD50-NBS1 (MRN) complex (Park et al., 2015), resulting in failure of DNA repair and permanent cell-cycle arrest. Thus, inhibition of CHK1 increases cellular dependence on autophagy to mitigate DNA damage, and conversely inhibition of autophagy contributes to further accumulation of damaged DNA.

In summary, oncogenic KRAS-driven PDAC cell growth is sensitive to the loss of genes important for regulating cell cycle, DDR, and macromolecule metabolism. Through our dissection of the signaling changes induced by CHK1i, we identified key signaling nodes that could exploited as therapeutic targets for combination inhibitor treatments for PDAC.

## LIMITATIONS OF THE STUDY

Currently, CHK1 inhibitors including prexasertib have stalled in clinical development, with only three ongoing trials (NCT04023669, NCT04032080, and NCT04095221). To address this limitation, we also evaluated the clinical candidate ATR inhibitor AZD6738/ceralasertib (ATRi). Although *CHEK1* genetic suppression was more effective at blocking PDAC growth than *ATR*, we did find that ATRi in combination with either ERK or CQ, like CHK1i, resulted in a more potent inhibition of PDAC growth. Thus, targeting either ATR or CHK1 in combination with other key PDAC pathways, ERK-MAPK and/or autophagy might be clinically beneficial. While CHK1 is a substrate of ATR, a proteomics analysis has revealed that ATR and ATM phosphorylate more than 700 proteins in response to DNA damage (Matsuoka et al., 2007). Therefore, further mechanistic studies would need to be done to verify the degree to which CHK1 inhibition mimics ATR inhibition in PDAC.

## STAR★METHODS

### RESOURCE AVAILABILITY

#### Lead contact

Further information and requests for resources and reagents should be directed to and will be fulfilled by the lead contact, Channing J. Der (cjder@med.unc.edu).

#### Materials availability

This study did not generate any unique reagents.

#### Data and code availability

RNA-seq data for PDAC cells treated with ERKi are publicly available (Bryant et al., 2019, PRJEB25806). Unnormalized counts and differential gene expression statistics are in Table S2. No unique code was generated for these analyses. CRISPR-Cas9 RSA and LogP values can be found in Table S3. No unique code was generated for these analyses.

### EXPERIMENTAL MODEL AND SUBJECT DETAILS

#### Cell lines

The patient-derived xenograft (PDX) human PDAC cell lines Pa01C, Pa02C, Pa04C, Pa14C, Pa16C, and Pa18C were supplied by Dr. Anirban Maitra (MD Anderson Cancer Center). The remaining PDAC cell lines and HEK293T were obtained from American Type Culture Collection (ATCC) and the HPNE cells have been previously described (Campbell et al., 2007). PDAC cell lines (Pa01C and Pa16C) stably expressing the Apple tagged trunc53BP1 were generated using the Apple-53BP1trunc plasmid, a gift from Ralph Weissleder (Addgene plasmid # 69531; RRID:Addgene_69531). PDAC cell lines expressing mCherry-EGFP-LC3B (Pa01C, Pa02C, and Pa16C) were generated as we have described previously (Bryant et al., 2019). Cell lines were maintained in either DMEM or RPMI 1640 and supplemented with 10% fetal bovine serum (FBS). All cell lines were verified negative for mycoplasma using the MycoAlert Mycoplasma Detection Kit (Lonza) and the PDAC cell line identities were verified by short tandem repeat analysis. Cell line working stocks were passage 4–5 and passaged for no more than 4 weeks so all experiments were performed in passage 15 or lower.

#### Patient-derived organoids

The hM1A and hT106 human PDAC patient-derived organoid cultures were provided by Dr. David Tuveson (Cold Spring Harbor Laboratory) and have been described previously (Tiriac et al., 2018). The PT3, PT6, PT8, PT11–2, and PT12 PDAC patient-derived organoid cultures were established by methods we described previously (Neal et al., 2018) and subsequently grown in the Matrigel dome method. PT3, PT6, and P8 have been previously described (Neal et al., 2018). Characteristics of these as well as the unpublished organoids PT11–2 and PT12 are summarized in Table S4. Organoids were cultured at 37°C, 5% CO_2_, in growth factor reduced Matrigel (Corning) domes in complete human feeding medium: Advanced DMEM/F12 (Thermo Fisher Scientific) based WRN conditioned medium (L-WRN (ATCC CRL-3276)), 1x B27 supplement (Thermo Fisher Scientific), 10 mM HEPES (Thermo Fisher Scientific), 0.01 μM GlutaMAX (Thermo Fisher Scientific), 10 mM nicotinamide (Sigma-Aldrich), 50 ng/mL hEGF (Peprotech), 100 ng/mL hFGF10 (Peprotech), 0.01 μM hGastrin I (TOCRIS), 500 nM A83–01 (TOCRIS), 1.25 mM and 1 mM N-acetylcysteine (Sigma-Aldrich) for hT105, hT106, hM1A, (Tiriac et al., 2018) PT3, PT6, PT8, PT11–2, and PT12 respectively. Medium for the latter five organoids additionally contained 10 μM SB202190 (Sigma), with 10.5 μM Y27632 (Selleck) added for the first two days after reseeding single cells. Organoids were routinely tested for mycoplasma and determined to be negative. Organoid lines were used between passage 10–20.

### METHOD DETAILS

#### siRNA “Druggable genome” screen with ERKi

The performance of this screen and the results have been previously published in full (Waters et al., 2021). For this study, *CHEK1* specific results were extracted from the full dataset. In brief four siRNA sequences for each gene from The Human Druggable Genome v3 siRNA Library (QIAGEN) were screened in 90 × 384-well plates. The siRNAs were printed individually into the assay plates (1 μL of 0.667 μM siRNA per well for a total of 9 ng siRNA) and each plate included negative control siRNAs (Non-Silencing, All-Star Non-Silencing, and GFP), and two positive control siRNAs (UBBs1 and All-Star Cell Death Control). Lipofectamine RNAiMax (Thermo Scientific) was used for transfection in serum free media. DMSO or various concentrations of ERKi was added following 24 h. Cell viability was measured using the CellTiter-Glo, following 96 h of drug treatment. The confirmation screen was performed the same way and hits were defined as genes that had at least two siRNAs above a threefold shift in the ERKi GI_50_ generated from the dose-response curve compared to controls. Details describing normalization, siRNA effectiveness, and transfection efficiency of the screen can all be found in the Star Methods and siRNAs in the supplemental information of Waters et al., 2021.

#### Lentivirus generation and infection

Lentivirus was generated as previously described (Martz et al., 2014). HEK293T cells (0.9 × 10^6^) were plated and incubated overnight in DMEM supplemented with 10% FBS in T25 flasks. Fugene 6 was used according to the manufactures recommendation to transfect the cells, 500 μl of Optimem was combined with 25 μl of Fugene 6, 3 μg of psPax, 1 μg of pMD2.G, and 4 μg of target construct per T25 flask. Cells were incubated overnight and the next day the media was replaced with DMEM supplemented with 20% FBS. After an additional 48 h the viral supernatant was removed and filtered through a 0.45 μm PES syringe filter (Nalgene). The filtered viral supernatant and polybrene (8 μg/ml) was used to infect target cells. Fresh media was given to the cells 12 h post infection and selection was initiated 48 h after transduction.

#### CRISPR/Cas9 “Druggable genome” and “ERK substrate” libraries

The design and cloning of the “druggable genome” library have been described previously (Ozkan-Dagliyan et al., 2020). In brief the CRISPR-Cas9 library targeted 2,240 genes relevant to cancer and 150 control genes where the essentiality was known (Hart et al., 2014). Five short guide RNA (sgRNA) were used to target each of the 2,390 genes. In addition, there were also 50 non-targeting sgRNA constructs, in total the library contained 12,000 sgRNAs. Further details about the generation of the library and complete list of guides and target genes can be found in the Star Methods and supplemental information in Ozkan-Dagliyan et al., 2020.

The ERK CRISPR sgRNA library was generated with the single vector pLentiCRISPRv2 (Sanjana et al., 2014). Putative ERK1/2 (ERK) substrate candidates were chosen from a curated list of validated or putative direct/indirect ERK substrates based on a compendium of data from 14 separate studies (Ünal et al., 2017). A total of 1308 UNIPROT notations were converted to ENTREZ IDs with the g:convert program. Thirteen unsuccessful gene ID conversions were converted to ENTREZ identifiers manually. Notably, we adopted relatively loose inclusion criteria to ensure coverage of all putative ERK substrates and to allow for the potential to identify secondary targets of ERK signaling. Additionally, 42 novel ERK substrates were included based on recent work identifying ERK substrates with multiplexed inhibitor beads technology. sgRNAs were designed from ENTREZ IDs with the Broad CRISPRko webtool (Doench et al., 2016). Five unique sgRNA inserts were synthesized for each target gene as previously described (Adhikari and Counter, 2018). In total, 1,223 putative ERK substrates were targeted by 6115 unique sgRNAs. As controls, sgRNAs were synthesized against the three RAS genes, the coding sequence of the *BRAF*, essential ribosomal genes, and 50 predicted non-targeting sequences as negative controls. A complete list of sgRNA guides used are in Table S3.

For both libraries the following protocol was used: sgRNA inserts were synthesized by custom array and cloned into the pLentiCRISPRv2 vector (Addgene vector # 52961) with the following protocol. The oligonucleotide pool was diluted 1:100 in water and amplified with Array:

Forward (5′- TAACTTGAAAGTATTTCGATTTCTTGGCTTTATATATCTTGTGGAAAGGACGAAACACCG - 3′)
Reverse (5′- ACTTTTTCAAGTTGATAACGGACTAGCCTTATTTTAACTTGCTATTTCTAGCTCTAA AAC - 3′)

primers according to the following protocol: 98°C for 30 s followed by 18 cycles of 98°C for 10 s, 63°C for 10 s, 72°C for 15 s, followed by an incubation at 72°C for 3 minutes. Inserts were purified with AxyPrep Magnetic Bead (1.8x) according to the manufacturer’s protocol (Axygen). The pLentiCRISPRv2 vector was cut with BsmbI and the 13 kb band was isolated with QIAquick Gel Extraction Kit according to the manufacturer’s protocol (QIAGEN). Array amplified sgRNAs were inserted into cut pLentiCRISPRv2 vector with a Gibson End Joining reaction. After Gibson assembly, 1 μL reactions were electroporated into *E. coli* 10G electrocompetent cells (Lucigen), recovered for one h in SOC medium, and spread on LB Ampicillin plates which were incubated overnight at 37°C. Dilution plates were counted to ensure sufficient library coverage and colonies were scraped in LB medium for extraction with a Plasmid Maxiprep kit (QIAGEN). Lentivirus was generated as describe above.

#### CRISPR-Cas9 virus titration

Virus titer was measured in each cell line evaluated. Each cell line was plated at a density of 4,000 cells/well in a 96-well clear bottom plate. The virus stock (25 μl per well) was added the next day. Serial dilutions of virus were done in triplicate starting with the undiluted virus and diluting by two-fold for eight points, additionally six wells were treated with media only. Polybrene was added to each well at a final concentration of 8 μg/ml. Plates were spun at 800*g* for 1 h and then incubated overnight. The following day medium in all wells was replaced. The replacement media contained 2 μg/ml puromycin in all but three of the non-treated wells. The cells were incubated for 2 days, stained with calcein, and counted using a MiniMax (Molecular Devices). The MOI was calculated using:

1/ln(1−% infectedcells)

The titer for each virus dilution per volume was calculated via a Poisson distribution:

$$p[k]=\frac{m^{k}e^{-m}}{k!}$$

The volume of stock required for a MOI = 0.2 transduction was calculated (a low MOI was used in order to reduce the chance of double construct transduction):

$$\mu l\;of\;\text{virus}\;=\frac{\left(\frac{\text{cells}}{\text{well}}\right)*0.2}{\;\text{Titer}\;\left(\frac{IFU}{\mu l}\right)}$$

#### CRISPR-Cas9 screen

Cells were plated at a density of 1×10^6^ cells per well in 25 × 6-well plates. The appropriate amount of virus and polybrene (8 μg/ml) and plates were spun at 800*g* for 1 h. The following day the transduction media was replaced with fresh media containing puromycin (2 μg/ml) and incubated for two days. Cells were trypsinized and plated on 500 cm^2^ plates, also 10^7^ cells were collected for a day 0 time point. Cells were allowed to proliferate for an additional 7 days and then trypsinized. At this time point 10^7^ cells were collected for the day 7 post-infection time point. The remaining cells were divided into either vehicle or treatment plates. For each condition four replicates were done with 10^7^ cells per replicate. Cells were continuously cultured in the presence of vehicle or treatment drug for another 4 weeks; fresh media and vehicle/drug was added every 4 days. Cells were not allowed to reach confluency and each replicate was split independently maintaining at least 10^7^ cells per replicate or 1000x the coverage of the library. At 14 and 28 days, post treatment 10^7^ cells from each replicate were collected. Collected cell pellets were washed with phosphate-buffered saline (PBS) and stored at −80°C. DNA was extracted with DNeasy Blood & Tissue Kit (QIAGEN) and prepared for sequencing. Following sample preparation and DNA purification via ethanol precipitation, samples were run in two subsequent PCR reactions as we have described previously (Ozkan-Dagliyan et al., 2020). Following the second reaction the entire reaction was gel purified using a Gel Extraction Kit (QIAGEN) and cleaned up via ethanol precipitation. Sequencing was performed on an Illumina NextSeq 500 with 75 bp single end reads.

#### siRNA transfections

Cells were reverse transfected using RNAiMax and 10 pM of siRNA in Optimem. RNAiMax was added to Optimem and allowed to equilibrate for 5 min at room temperature. The siRNAs were then added to the Optimem + RNAiMax mixture and incubated for 20 min. The siRNA Optimem RNAiMax mixture was added dropwise to the cells and incubated until collection time. For all siRNA, a non-targeting siRNA control was used, and knockdown of target gene was confirmed in parallel by either RT-qPCR or immunoblotting. Additionally, at least two independent siRNAs were utilized to target all genes of interest. For combined siRNA and drug studies, the cells were transfected the evening prior and then drugged the following day. All siRNA sequences Table S3.

#### Colony forming assays

Cells were plated in 6-well plates at 4,000 cells per well on day 0 in 2 mL DMEM supplemented with 10% FBS. The following day the medium was changed and replaced with media containing the indicated amount of drug or equivalent concentration of vehicle (DMSO). Cells were allowed to grow for 8–10 days, washed with PBS, and then stained with crystal violet in formaldehyde. Following staining the plates were submerged in a bath of DDH_2_O and allowed to dry. Plates were imaged using a Typhoon^™^ FLA 7000 biomolecular imager. FIJI was used analyze % cell coverage of the plate surface all wells were standardized to their respective DMSO. The mean and standard deviations were determined between three to four biological replicates.

#### Proliferation assays

Cells were plated in 96-well plates (1,000–2,000 cells per well depending on the cell line) and grown for 24 h prior to drug treatment. Drugs were added using Tecan D300e digital dispenser. Cells were then incubated for 5 days after which they were imaged using a SpectraMax i3X multimode detection platform (Molecular Devices). Cells were live counted via labeling with calcein AM.

#### Organoid proliferation assays

PDAC organoids were dissociated and 3 × 10^3^ cells were seeded in 150 μl of 90% human organoid feeding media supplemented with 10.5 μM Y27632 (Selleckchem) and 10% growth factor reduced Matrigel (Corning) onto 96-well clear flat bottom plates (Corning) coated with poly(2-hydroxyethyl methacrylate) (Sigma-Aldrich). On the following day, organoids were drugged with CHK1i (prexasertib, 2 to 32 nM), ERKi (SCH772984, 0.008 to 2 μM) and chloroquine (3.25 μM) using a Tecan D300e digital dispenser. Ten days after drugging, organoids were imaged with a Molecular Devices SpectraMax i3x MiniMax 300 imaging cytometer. After image acquisition, organoid viability was assessed with the CellTiter-Glo 3D Cell Viability Assay (Promega) on a SpectraMax i3x plate reader, according to the manufacturer’s protocol.

#### CellTox cytotoxicity assays

Cells were plated at high densities in 96-well plates (8,000–10,000 cells per well depending on the cell line) and grown for 24 h prior to drug treatment. Drugs were added using a Tecan D300e digital dispenser at the indicated concentrations and then incubated for 72 h. CellTox Green was added to the cells according to manufactures recommendation 3 h prior to endpoint. Fluorescence levels were measured using the SpectraMax i3X (Ex. 485 nm, Em. 520 nm). All samples were standardized to their control value and the mean log2 fold change was calculated from four biological replicates.

#### Flow cytometry apoptosis and cell cycle assays

Apoptosis analyses were performed with the TACs Annexin V-FITC kit (Trevigen) according to the manufacturer’s recommendations. In brief, detached cells from both supernatant and following trypsin recovery were centrifuged at 300 × g for 5 min. Cells were washed with ice cold PBS and subsequently incubated in Annexin V incubation reagent (1% Annexin V-FITC, 1x propidium iodide solution, 1x calcium-containing binding buffer) at room temperature, in the dark, for 15 min and then diluted 1:5 in 1x binding buffer. Cells were analyzed using a BD LSRFortessa flow cytometer and collected using FACSDiva v8.0.1. For each sample > 20,000 cells were collected, to avoid collection of debris we used a scatterplot of side scatter area (SSC-A) (y) versus forward scatter area (FSC-A) (x) to collect only intact cells. Cytobank was used to determine apoptosis percentages, this was done by plotting propidium iodide area (ECD-A) (y) versus FITC area (FITC-A). Gates were established using the vehicle control samples and maintained for the analysis of all associated samples. Apoptosis was designated to be the cells in the top right quadrant (high ECD-A and high FITC-A) and lower right quadrant (low ECD-A and high FITC-A). The percent apoptosis in the vehicle control was subtracted from all samples for each biological replicate. The mean and standard deviation was calculated for n = 3 independent biological replicates. Statistical comparisons used for analysis are stated in the respective figure legends. Cell cycle analyses were performed with FCS Express. An FSC-A (x) versus FSC-H (y) dot plot was used to establish a “singlets” gate. Singlets were then analyzed via a histogram for ECD-A content prior to employing a Multicycle algorithm to analyze cell cycle.

#### Quantitative reverse transcriptase PCR

Total RNA was extracted using a RNeasy kit (QIAGEN) and reverse-transcription was done using the High Capacity RNA-to-cDNA kit (Thermo Fisher). Real-time quantitative PCR was done on a QuantStudio 6 Flex (Thermo Fisher) using TaqMan PCR (Applied Biosciences). FAM dye labeled probes (Thermo Fisher) were used against *KRAS*, *CHEK1*, *MYC*, and *RIF1* and a VIC dye labeled probe was against *ACTB*, as a house-keeping control. Delta-delta CT was calculated for each sample. All samples were run as technical duplicates which were then standardized to their respective NS or vehicle treated controls. Means and standard deviations were calculated from the standardized values of three to four biological replicates.

#### Immunoblot analyses

Cells were washed with cold PBS and scraped into lysis buffer (20 mM HEPES-KOH, pH 7.8, 50 mM KCl, 100 mM NaCl, 1 mM EGTA, 1% NP40) supplemented with protease (Roche) and phosphatase (Sigma-Aldrich) inhibitors and incubated 10 min on ice. Samples were centrifuged at 8,000 rpm, 4°C, for 10 min and cleared lysate was transferred to a new tube. Protein concentration was determined using a Bradford assay (Bio-Rad) from cleared lysates. Equal amounts of protein per sample were loaded into gels and standard immunoblotting procedures were utilized.

#### Reverse phase protein array (RPPA)

Pa01C, Pa02C, Pa14C, Pa16C, MIA PaCa-2 and PANC-1 cells were plated onto 6-well plates. The next day cells were treated with vehicle, prexasertib (CHK1i) (15 nM), SCH772984 (ERKi) (200 nM), or prexasertib + SCH772984 (CHK1i + ERKi) (15 nM + 200 nM) for 4, 24, or 72 h. At the appropriate time point cell lysates were prepared as previously described (Baldelli et al., 2017; Pierobon et al., 2017). All treatments were done in biological quadruplets. The protein concentration was determined using Coomassie Protein Assay Reagent kit (Thermo Fisher Scientific) as described by the manufacturer. Cell protein was diluted to 0.5 mg/ml in 2x tris-glycine SDS Sample buffer (Life Technologies) with 5% β-mercaptoethanol and boiled for 8 min and stored at −80°C until put on the array. Cell lysates were immobilized using an Aushon 2470 automated system (Aushon BioSystems) as previously described in (Baldelli et al., 2017). Samples were printed in technical replicates (n = 3) along with reference standards used as internal controls. To quantify the amount of total protein present in each sample, selected arrays were stained with Sypro Ruby Protein Blot Stain (Molecular Probes) following manufacturing instructions. Remaining arrays were treated with Reblot Antibody Stripping solution (MilliporeSigma) for 15 min at room temperature, followed by two washes with PBS, and incubated for 4 h in I-block (Tropix) prior to immunostaining (Baldelli et al., 2017). Immunostaining was performed using an automated system (Dako Cytomation) as previously described (Baldelli et al., 2017). Each array was probed with one antibody targeting the proteins of interest. Arrays were probed with a total of 152 antibodies (Table S1). Each antibody was validated for its specificity as previously described (Signore et al., 2017). Biotinylated antirabbit (Vector Laboratories, Inc.) or anti-mouse secondary antibodies (DakoCytomation) coupled with a commercially available tyramide-based avidin/biotin amplification system (Catalyzed Signal Amplification System; DakoCytomation) and the RDye 680RD Streptavidin (LI-COR Biosciences) fluorescence dye were used for the signal amplification and detection. Images for Sypro Ruby and antibody-stained slides were acquired using a Tecan laser scanner (TECAN) and images were analyzed using the commercially available software MicroVigene Version 5.1.0.0 (Vigenetech) as previously described (Pin et al., 2014).

#### Immunofluorescence and imaging

All cells were plated on glass bottom dishes (MatTEK Corporation) for imaging. RIF1, RAD51, endogenous 53BP1, mApple-53BP1trunc and γH2AX were imaged in fixed cells using an IX-81 or EVOS M7000 wide-field microscope with a 63X, 1.4 NA objective. Colocalization of endogenous 53BP1 with γH2AX and mCherry-EGFP-LC3B expressing cells was performed on a Zeiss 700 confocal microscope with a 63x, 1.4 NA objective. The mCherry-EGFP-LC3B cells were imaged live. Cells were fixed with 4% formaldehyde for 20 min at room temperature. For RIF1, 53BP1, RAD51, and γH2AX staining the cells were permeabilized with 0.1% Triton for 5 min, rinsed with PBS, and blocked using 3% BSA-PBS for 1 h at room temperature. The cells were incubated in antibodies targeting RIF1, 53BP1, RAD51, and γH2AX (1:200 in 3% BSA-PBS) overnight at 4°C or at room temperature for 2 h. The 53BP1 antibody was pre-conjugated with Alexa-647. For the remainder of the antibodies, cells were washed with PBS followed by incubation in Alexa 488 and Alexa-561 secondary antibodies. To visualize nuclei, cells were stained with DAPI (1:10,000) for 5–10 min in PBS and subsequently rinsed with PBS.

### QUANTIFICATION AND STATISTICAL ANALYSIS

#### Statistical analyses

All statistical analyses unless otherwise stated were done using GraphPad Prism version 8.3.0. Prior to statistical analyses normality of data was assessed using a D’Agostino-Pearson normality test. To compare a single treatment to a control an unpaired t test was used. To analyze experiments with multiple treatments or conditions a one-way or two-way ANOVA for with either a Dunnett or Tukey multiple comparison test was used to compare every treatment mean to a defined control mean. To compare pre-selected means a Sidak multiple comparison test was used. For ERK inhibitor RNA-seq data a dispersion corrected moderated t test was implemented in limma. For all tests *p < 0.05, **p < 0.01, ***p < 0.001, and ****p < 0.0001. The tests utilized and replicate numbers are stated in the figure legends. Except for the number of cells/nuclei analyzed for imaging experiments, those are listed in the image analyses section below.

#### CRISPR-Cas9 screens

Sample barcode and sgRNA sequence counts were deconvolved to obtain counts for each construct of every gene in the “druggable” genome library (Shalem et al., 2014). The counts for each construct were normalized to the total count from the same sample and then the mean across replicates was calculated. The drug-treated sample counts were compared to vehicle-treated samples in order to determine the relative change for each sgRNA upon drug treatment. For essential gene analysis, samples collected nine days after puromycin selection were compared to samples that were collected before puromycin exposure. A ranked gene list was generated based on p values determined by the redundant siRNA activity (RSA) method, a probability-based algorithm analyzing the collective activity of multiple siRNAs per gene (König et al., 2007). Reactome pathway analysis of the ranked gene list through STRING (Search Tool for the Retrieval of Interacting Genes/Proteins) (Szklarczyk et al., 2019) identified enriched pathways sensitizing PDAC cells to drug treatment.

#### Analyses of growth assays

To analyze the colony forming proliferation assays we used FIJI to calculate the percent coverage of each well. This was done by first generating a binary mask of the stained area and using the generated mask to calculate the percent coverage of the total well area. The relative percent coverage was determined by standardizing to the vehicle treatment well which was set to 100% for each biological replicate. The mean and standard deviation was calculated for each biological replicate and graphed against the log concentration GraphPad Prism version 8.3.0. Specifically, three-parameter drug response curves were generated using via the following equation:

$$Y=\text{ Bottom }+\left(\frac{\text{ Top }-\text{ Bottom }}{1+10^{\left(\text{X}-\text{Log}(\text{IC}50)\right)}}\right)$$

Representative images of each treatment are shown for each condition.

For the calcein proliferation assays and CellTiter-Glo organoid assays the percentage growth was calculated by normalizing treated values to their respective control samples, which were set to 100%. GraphPad Prism version 8.3.0 was used to generate three-parameter drug response curves as described above. From the generated curves we calculated the mean GI_50_ from three to four biological replicates. A one-way ANOVA with a Dunnett’s multiple comparison test was used to calculate significance for single versus dual inhibitor treatment. For the triple combinations a two-way ANOVA and Tukey’s multiple comparison test was used.

Heatmaps for cell line sensitivity to individual drugs were calculated using the mean GI_50_. The heatmaps for organoid growth sensitivities to single and combinations of drugs were generated using the median percent growth from three biological replicates. Both cell line and organoid growth heatmaps were generated using GraphPad Prism version 8.3.0.

#### Bliss and kill effect

The kill effect was calculated by subtracting the normalized viability from 1 and multiplying that by 100. All graphs show the mean of three to four biological replicates. Bliss calculation for the double and triple combinations was done as described previously (Foucquier and Guedj, 2015), using the following equations:

$$\text{bliss}\;=\frac{\;\text{Expected}}{\;\text{Observed}}$$

$$\text{Double Expected }=\left(M_{\text{A}}+M_{\text{B}}\right)-\left(M_{\text{A}}\times M_{\text{B}}\right)$$

$$\text{Triple Expected }=\left(M_{\text{A}}+M_{\text{B}}+M_{\text{C}}\right)-\left(M_{\text{A}}\times M_{\text{B}}\right)-\left(M_{\text{A}}\times M_{\text{C}}\right)-\left(M_{\text{B}}\times M_{\text{C}}\right)+\left(M_{\text{A}}\times M_{\text{B}}\times M_{\text{C}}\right)$$

M = Mortality = 1-normalized viability

The values used in the bliss calculation were generated from the mean of three to four biological replicates. All Bliss synergy scores for all combinations have been provided in Table S4. Values which gave negative scores are annotated by an “X.” Negative scores are the result of one drug causing an increase in growth at a specific dose and thus resulting in a misleading value.

#### TCGA analyses

The TCGA Pancreatic adenocarcinoma (PAAD) clinical data were retrieved from supplemental table S1 (Liu et al., 2018). IlluminaHi-Seq RNASeqV2 datasets were accessed using UCSCXenaTools R package (version 1.3.3). Only patient samples defined as “high purity” (Raphael et al., 2017) were used for analysis. The upper quantile (> 75%) of mRNA counts were defined as high expression compared with low expression and used to fit to a Cox proportional hazard model using the survival R package (version 3.1–12). The p value was calculated from a Wald test of the Cox regression model.

#### Image analyses

Endogenous 53BP1 and 53BP1-mApple foci analyses were done using FIJI. Individual nuclei were circled via the DAPI channel and the number of foci was determined via the Find Maxima function. Analysis of γH2AX intensity was also determined using FIJI. As before stacks were used to generate a composite γH2AX images. Nuclei were then circled via the DAPI channel and the integrated intensity in the γH2AX channel was determined. For all assays two to three different biological preparations were done for each condition. The relative integrated intensity of γH2AX was calculating the median value of the vehicle treated sample from each biological preparation. All values were then divided by their respective vehicle value. No difference was found between the biological preparations for endogenous 53BP1, trunc53BP1-mApple foci or relative γH2AX intensity, therefore all nuclei were grouped together for statistical analysis. RIF1 foci were quantified by calculating the % nuclei containing foci at least five fields of view from two biological preparations for each condition. The same approach was used to calculate the % nuclei with no γH2AX, foci-γH2AX, or pan-γH2AX as well as for % nuclei containing ≥ 3 RAD51 foci. For all assays two to three biological preparations were prepared. Sample sizes for each condition are as follows: Pa01C and Pa16C γH2AX intensity, n = 150 nuclei for all conditions from three biological replicates; trunc-53BP1-mApple n = the following number of nuclei per condition, Pa01C (DMSO = 208, 8 nM = 194, 32 nM = 163, DMSO+NCS = 211, 8 nM+NCS = 171, 32 nM+NCS = 173), Pa16C, (DMSO = 235, 8 nM = 244, 32 nM = 242, DMSO+NCS = 234, 8nM+NCS = 231, 32 nM+NCS = 219) from three biological replicates, Pa16C+/−ERKi all conditions n = 100 nuclei from two biological replicates, Pa16C+/−RIF1 knockdown all conditions n = 50 nuclei from two biological replicates; endogenous 53BP1 n = the following number of nuclei per condition, Pa01C (DMSO = 224, 8 nM = 227, 32 nM = 224, DMSO+NCS = 232, 8 nM+NCS = 218, 32 nM+NCS = 227) and Pa16C, (DMSO = 266, 8 nM = 233, 32 nM = 255, DMSO+NCS = 267, 8nM+NCS = 224, 32 nM+NCS = 258) from two biological replicates; % cells for γH2AX total nuclei, Pa01C and Pa16C all conditions = 50 nuclei from three biological replicates; % cells with RIF1 foci number of nuclei analyzed from 10 frames and two biological replicates, Pa01C (0 nM = 139 and 8 nM = 131) and Pa16C (0 nM = 158 and 32 nM = 143); for % cells with RAD51 foci number of nuclei analyzed from 10 frames and two biological replicates, Pa16C (DMSO = 667, 8 nM = 613, 32 nM = 443, DMSO+NCS = 504, 8nM+NCS = 575, 32 nM+NCS = 413). Quantification of autophagic flux was performed as we described previously (Bryant et al., 2019). In brief images were acquired in line mode in order to reduce movement of autophagosomes between channel acquisition. The autophagic index is the ratio of the total fluorescence of mCherry positive vesicles to the total fluorescence of EGFP positive vesicles. For each condition three different biological replicates were evaluated.

#### DepMap analyses

We analyzed existing data from the Cancer Dependency Map: DepMap 20Q4) (Tsherniak et al., 2017). Median dependency scores were calculated for each kinase specifically from the PDAC samples using both the shRNA (McFarland et al., 2018) and CRISPR-Cas9 data (Dempster et al., 2019; Meyers et al., 2017).

#### RNA sequencing analysis

In this study, we reanalyzed RNA-seq data for control and 24 h 1 μM ERK inhibitor (SCH772984, ERKi) treated PDAC cells (PRJEB25806) (Bryant et al., 2019) and additional untreated PDAC cells (PRJEB38063) (Ozkan-Dagliyan et al., 2020). The cell lines examined included HPAC, HPAF-II, Pa01C, Pa04C, Pa14C, PANC-1, and SW1990. Basic quality control, including adaptor removal and quality trimming, was conducted via TrimGalore v0.4.5 (Krueger, 2012). The STAR v2.6.0 (Dobin et al., 2013) sequence aligner was used with genome version GRCh38.p12 and Gencode v30 annotation. Salmon v1.3.0 (Patro et al., 2017) was used for transcriptome quantitation and data were imported and manipulated in R via tximport (Soneson et al., 2015) and biomaRt (Durinck et al., 2005) was used for transcript annotation. Differential expression comparisons were conducted using the VOOM weighted (Law et al., 2014) empirical Bayes linear model of the covariates of experiment, time (h) + 1, and ERKi as implemented in limma (Ritchie et al., 2015). The fgsea (Korotkevich et al., 2021) package was used to calculate normalized enrichment statistics for DNA-damage related gene sets from the molecular signature database MsigDB (Liberzon et al., 2015).

#### RPPA analysis

Antibody intensity values were imported into R (version 3.5.2), values were log2 transformed, and the fold change over the respective vehicle value was calculated for each antibody. Heatmaps depict the median value for each cell line, time point, and treatment.

## Supplementary Material

1

2

3

4

5

6

## Figures and Tables

**Table:** KEY RESOURCES TABLE

REAGENT or RESOURCE	SOURCE	IDENTIFIER
Antibodies		
Rabbit monoclonal anti-phospho-CHK1 (133D3) (pCHK1) (Ser345)	Cell Signaling Technology	Cat # 2348; RRID: AB_331212
Mouse monoclonal anti-CHK1 (2G1D5) (CHK1)	Cell Signaling Technology	Cat # 2360; RRID: AB_2080320
Rabbit monoclonal anti-phospho-H2AX (20E3) (γH2AX) (Ser139)	Cell Signaling Technology	Cat # 9718; RRID: AB_2118009
Rabbit monoclonal anti-phospho-RB (D59B7) (pRB) (S780)	Cell Signaling Technology	Cat # 8180; RRID: AB_10950972
Rabbit monoclonal anti-phospho-Cdc2 (pCDK1) (Tyr15)	Cell Signaling Technology	Cat # 9111; RRID: AB_331460
Rabbit monoclonal anti-WEE1 (D10D2) (WEE1)	Cell Signaling Technology	Cat # 13084; RRID: AB_2799665
Rabbit monoclonal anti-c-MYC (D84C12) (MYC)	Cell Signaling Technology	Cat # 5605; RRID: AB_1903938
Rabbit monoclonal anti-phospho-AMPKα (D4D6D) (pAMPK) (Thr172)	Cell Signaling Technology	Cat # 50081; RRID: AB_2799368
Rabbit monoclonal anti-ATR (E1S3S) (ATR)	Cell Signaling Technology	Cat # 13934; RRID: AB_2798347
Rabbit monoclonal anti-ATM (D2E2) (ATM)	Cell Signaling Technology	Cat # 2873; RRID: AB_20662659
Rabbit monoclonal anti-phospho MEK1/2 (166F8) (pMEK) (Ser221)	Cell Signaling Technology	Cat # 2338; RRID: AB_490903
Rabbit polyclonal anti-phospho-p44/42 MAPK (pERK) (Thr202/Tyr204)	Cell Signaling Technology	Cat # 4370; RRID: AB_2315112
Rabbit polyclonal anti-p44/42 MAPK (ERK)	Cell Signaling Technology	Cat # 9102; RRID: AB_330744
Rabbit polyclonal anti-phospho-AKT (pAKT) (Ser473)	Cell Signaling Technology	Cat # 9271; RRID: AB_329825
Rabbit polyclonal anti-DUSP6/MKP3 (DUSP6)	Cell Signaling Technology	Cat # 39441; RRID: AB_2799156
Rabbit polyclonal anti-RAD51 (RAD51)	Thermo Fisher Scientific	Cat # PA5-27195; RRID: AB_2544671
Mouse monoclonal anti-β-actin	Sigma-Aldrich	Cat # A5441; RRID: AB_476744
Mouse monoclonal anti-vinculin	Sigma-Aldrich	Cat # V9131; RRID: AB_477629
Mouse monoclonal anti-KRAS	Sigma-Aldrich	Cat # WH0003845M1; RRID: AB_1842235
Mouse monoclonal anti-RIF1(B-3)	Santa Cruz	Cat # sc-515573
Mouse monoclonal anti-pan-AKT (40D4) (AKT)	Cell Signaling Technology	Cat # 2920; RRID: AB_1147620
Mouse monoclonal anti-phospho-H2AX (3F2) (γH2AX) (S139)	Thermo Fisher Scientific	Cat # MA1-2022; RRID: AB_559491
Mouse monoclonal anti-53BP1 (E-10) (53BP1) conjugated Alexa Fluor 647	Santa Cruz	Cat # sc-5158541
Goat anti-Mouse IgG Alexa Fluor 488	Thermo Fisher Scientific	Cat # A-32723; RRID: AB_2633275
Goat anti-Rabbit IgG Alexa Fluor 568	Thermo Fisher Scientific	Cat # A-11011; RRID: AB_143157
Goat anti-Rabbit IgG Alexa Fluor 488	Thermo Fisher Scientific	Cat # A-11008; RRID: AB_143165
Bacterial, virus strains, and plasmids		
DH5α	Thermo Fisher Scientific	Cat # 18258012
mApple 53BP1-trunc	Addgene	Plasmid #69531
Empty Vector	(Martz et al., 2014)	N/A
MYC WT	(Waters et al., 2021)	N/A
Chemicals, peptides, and recombinant proteins		
Human Druggable Genome siRNA Library v3	QIAGEN	(Waters et al., 2021)
SCH772984 (ERK1/2 inhibitor)	Provided by Merck	N/A
Prexasertib (LY2606368) (CHK1 inhibitor)	Selleckchem	Cat # S7178
AZD7762 (CHK1 inhibitor)	Selleckchem	Cat # S1532
Ceralasertib/AZD6738	Selleckchem	Cat # S7693
Chloroquine diphosphate	Sigma-Aldrich	Cat # 6628
Ipatasertib (AKT inhibitor)	Selleckchem	Cat # S2808
Trametinib (MEK1/2 inhibitor)	Selleckchem	Cat # S2673
Neocarzinostatin	Sigma-Aldrich	Cat # N9162
Cycloheximide (translation inhibitor)	Sigma-Aldrich	Cat # C4859
MG132 (proteasome inhibitor)	Sigma-Aldrich	Cat # M7449
DAPI	Thermo Fisher Scientific	Cat # D3571
RNase A	Thermo Fisher Scientific	Cat # EN0531
Propidium iodide	Thermo Fisher Scientific	Cat # P3566
2-hydroxypropyl-b-cyclodextrin	Sigma-Aldrich	Cat # H107
Matrigel® Growth Factor Reduced Basement Membrane Extract, Phenol Red-free, LDEV-free	Corning	Cat # 356231
Matrigel® Basement Membrane Matrix, Phenol Red-free, LDEV free	Corning	Cat # 356237
XeonLight D-Luciferin -K+	Perkin Elmer	Cat # 122799
Critical commercial assays		
TACS Annexin V-FITC *in situ* apoptosis detection kit	Trevigen, Inc.	Cat # 4830
CellTiter-Glo Luminescent Cell Viability Assay	Promega	Cat # G7570
CellTiter-Glo 3D Cell Viability Assay	Promega	Cat # G9683
Oligonucleotides		
*MYC* qPCR probe	Thermo Fisher Scientific	HS00153408_m1
*β-actin* qPCR probe	Thermo Fisher Scientific	4310881E-1711049
KRAS qPCR probe	Thermo Fisher Scientific	Hs00364284_g1
*CHEK1* qPCR probe	Thermo Fisher Scientific	Hs00967506_m1
*RIF1* qPCR probe	Thermo Fisher Scientific	HS04962410_m1
Software and algorithms		
GraphPad Prism version 8.0	GraphPad	https://www.graphpad.com/scientific-software/prism/
FCS Express version 7.0	De Novo Software	https://denovosoftware.com/
Cytobank version 7.3.0	(Kotecha et al., 2010)	https://www.cytobank.org/
STRING	(Szklarczyk et al., 2019)	https://string-db.org
R (version 3.5.1)		https://www.R-project.org/
Experimental models: Cell lines		
Human: Pa01C (pancreatic ductal adenocarcinoma)	(Jones et al., 2008)	N/A
Human: Pa02C (pancreatic ductal adenocarcinoma)	(Jones et al., 2008)	N/A
Human: Pa04C (pancreatic ductal adenocarcinoma)	(Jones et al., 2008)	N/A
Human: Pa14C (pancreatic ductal adenocarcinoma)	(Jones et al., 2008)	N/A
Human: Pa16C (pancreatic ductal adenocarcinoma)	(Jones et al., 2008)	N/A
Human: AsPC-1 (pancreatic ductal adenocarcinoma)	ATCC	Cat # CRL-1682; RRID: CVCL_0152
Human: HPAC (pancreatic ductal adenocarcinoma)	ATCC	Cat # CRL-2119; RRID: CVCL_3517
Human: HPAF-II (pancreatic ductal adenocarcinoma)	ATCC	Cat# CRL-1997; RRID: CVCL_0313
Human: MIA PaCa-2 (pancreatic ductal adenocarcinoma)	ATCC	Cat # CRL-1420; RRID: CVCL_0428
Human: PANC-1 (pancreatic ductal adenocarcinoma)	ATCC	Cat # CRL-1469; RRID: CVCL_0480
Human: SW 1990 (pancreatic ductal adenocarcinoma)	ATCC	Cat # CRL-2172; RRID: CVCL_1723
Human: BxPC-3	ATCC	Cat # CRL-1687; RRID: CVCL_0186
Human: PATC-153	(Yu et al., 2019)	N/A
Mouse: NIH/3T3	ATCC	Cat # CRL-1658; RRID: CVCL_0594
Human: HEK293T	ATCC	Cat # CRL-3216; RRID: CVCL_0063
Human: hTERT-HPNE E6/E7/KRAS G12D	(Campbell et al., 2007)	N/A
Human: hTERT-HPNE E6/E7	(Campbell et al., 2007)	N/A
Human: hTERT-HPNE	(Campbell et al., 2007)	N/A
Experimental models: Organoids		
Human: hT105	(Tiriac et al., 2018)	N/A
Human: hT106	(Tiriac et al., 2018)	N/A
Human: hM1A	(Tiriac et al., 2018)	N/A
PT12	This paper	N/A
PT11-2	This paper	N/A
PT3	(Neal et al., 2018)	N/A
PT6	(Neal et al., 2018)	N/A
PT8	(Neal et al., 2018)	N/A

## Highlights

- Pancreatic cancer cells are dependent on *CHEK1* and DNA damage response genes
- CHK1 inhibition causes compensatory ERK and autophagy activation
- Concurrent CHK1 and ERK inhibition causes synergistic loss of MYC
- Concurrent CHK1, ERK, and autophagy inhibition synergistically suppresses growth
