# Supplementary material for: CHK1 protects oncogenic KRAS-expressing cells from DNA damage and is a target for pancreatic cancer treatment
Source: Cell Rep. Author manuscript; Available in PMC 2021 Dec 11. (PMC8665414; doi:10.1016/j.celrep.2021.110060)
Supplement: 1 [file NIHMS1760956-supplement-1.pdf]

Cell Reports, Volume *37*

Supplemental information

CHK1 protects oncogenic KRAS-expressing cells from DNA damage and is a target for pancreatic cancer treatment

Jennifer E. Klomp, Ye S. Lee, Craig M. Goodwin, Björn Papke, Jeff A. Klomp, Andrew M. Waters, Clint A. Stalnecker, Jonathan M. DeLiberty, Kristina Drizyte-Miller, Runying Yang, J. Nathaniel Diehl, Hongwei H. Yin, Mariaelena Pierobon, Elisa Baldelli, Meagan B. Ryan, Siqi Li, Jackson Peterson, Amber R. Smith, James T. Neal, Aaron K. McCormick, Calvin J. Kuo, Christopher M. Counter, Emanuel F. Petricoin III, Adrienne D. Cox, Kirsten L. Bryant, and Channing J. Der

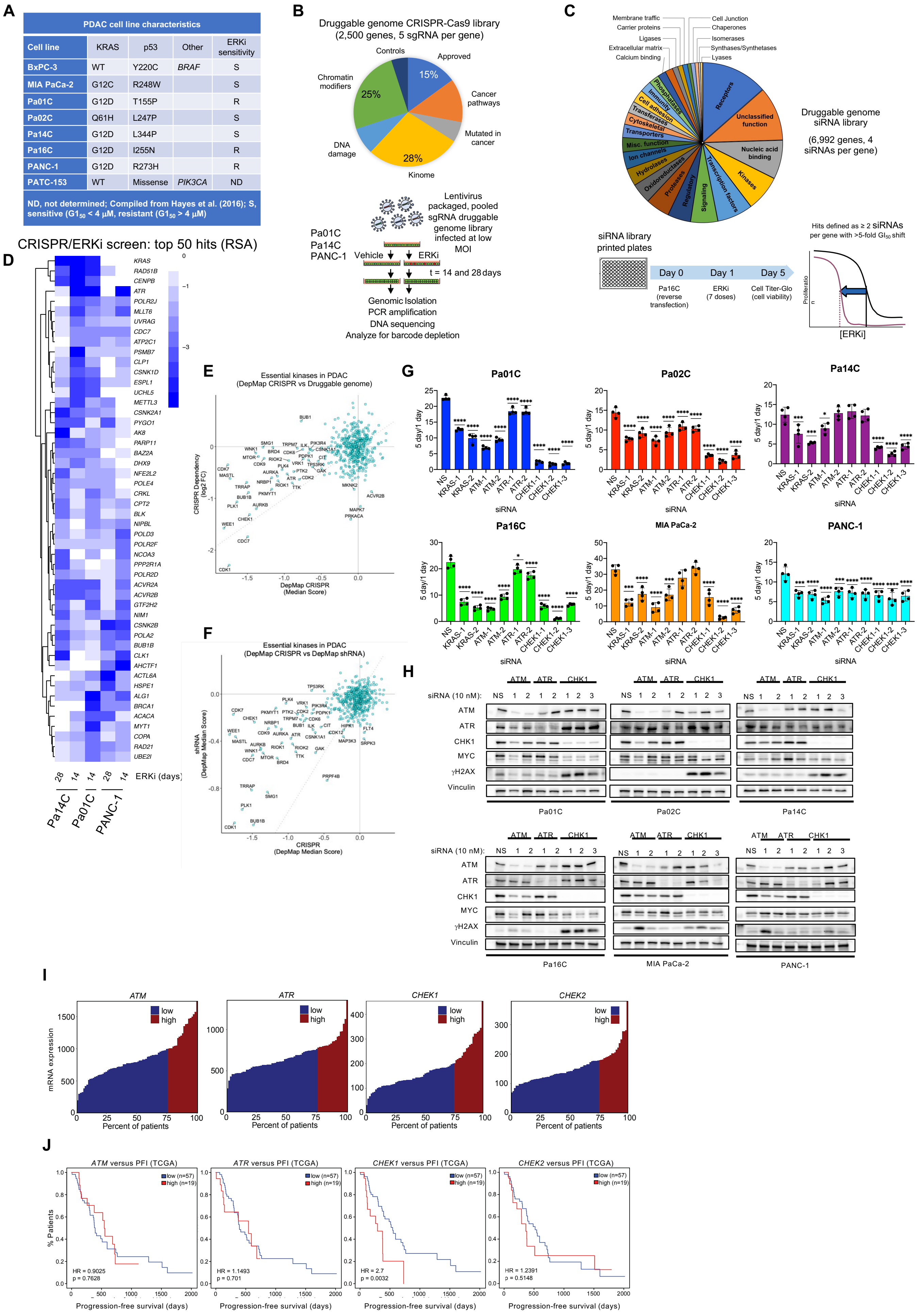
A
PDAC cell line characteristics

Cell line	KRAS	p53	Other	ERKi sensitivity
BxPC-3	WT	Y220C	*BRAF*	S
MIA PaCa-2	G12C	R248W		S
Pa01C	G12D	T155P		R
Pa02C	Q61H	L247P		S
Pa14C	G12D	L344P		S
Pa16C	G12D	I255N		R
PANC-1	G12D	R273H		R
PATC-153	WT	Missense	*PIK3CA*	ND

ND, not determined; Compiled from Hayes et al. (2016); S, sensitive (GI_{50} < 4 μM, resistant (GI_{50} > 4 μM)
B
Druggable genome CRISPR-Cas9 library (2,500 genes, 5 sgRNA per gene)
Controls
Approved
15%
Chromatin modifiers
25%
Cancer pathways
Mutated in cancer
DNA damage
28%
Kinome
Lentivirus packaged, pooled sgRNA druggable genome library infected at low MOI
Pa01C
Pa14C
PANC-1
Vehicle
ERKi
t = 14 and 28 days
Genomic Isolation
PCR amplification
DNA sequencing
Analyze for barcode depletion
C
Membrane traffic
Carrier proteins
Ligases
Extracellular matrix
Calcium binding
Cell Junction
Chaperones
Isomerases
Synthases/Synthetases
Lyases
Receptors
Unclassified function
Nucleic acid binding
Kinases
Transcription factors
Signaling
Regulatory
Proteases
Oxidoreductases
Hydrolases
Ion channels
Misc. function
Transporters
Cytoskeletal
Transferases
Cell adhesion
Immunity
Phosphatases
Druggable genome siRNA library
(6,992 genes, 4 siRNAs per gene)
siRNA library printed plates
Day 0
Day 1
Day 5
Pa16C (reverse transfection)
ERKi (7 doses)
Cell Titer-Glo (cell viability)
Hits defined as ≥ 2 siRNAs per gene with >5-fold GI_{50} shift
Proliferation
[ERKi]
D
CRISPR/ERKi screen: top 50 hits (RSA)
KRAS
RAD51B
CENPB
ATR
POLR2J
MLLT6
UVRAG
CDC7
ATP2C1
PSMB7
CLP1
CSNK1D
ESPL1
UCHL5
METTL3
CSNK2A1
PYGO1
AK8
PARP11
BAZ2A
DHX9
NFE2L2
POLE4
CRKL
CPT2
BLK
NIPBL
POLD3
POLR2F
NCOA3
PPP2R1A
POLR2D
ACVR2A
ACVR2B
GTF2H2
NIM1
CSNK2B
POLA2
BUB1B
CLK1
AHCTF1
ACTL6A
HSPE1
ALG1
BRCA1
ACACA
MYT1
COPA
RAD21
UBE2I
0
−1
−3
28
14
14
28
14
ERKi (days)
Pa14C
Pa01C
PANC-1
E
Essential kinases in PDAC (DepMap CRISPR vs Druggable genome)
CRISPR Dependency (log2 FC)
DepMap CRISPR (Median Score)
F
Essential kinases in PDAC (DepMap CRISPR vs DepMap shRNA)
shRNA (DepMap Median Score)
CRISPR (DepMap Median Score)
G
Pa01C
Pa02C
Pa14C
Pa16C
MIA PaCa-2
PANC-1
5 day/1 day
NS
KRAS-1
KRAS-2
ATM-1
ATM-2
ATR-1
ATR-2
CHEK1-1
CHEK1-2
CHEK1-3
siRNA
H
siRNA (10 nM):
NS
ATM
ATR
CHK1
MYC
γH2AX
Vinculin
Pa01C
Pa02C
Pa14C
Pa16C
MIA PaCa-2
PANC-1
I
ATM
ATR
CHEK1
CHEK2
low
high
mRNA expression
Percent of patients
J
ATM versus PFI (TCGA)
ATR versus PFI (TCGA)
CHEK1 versus PFI (TCGA)
CHEK2 versus PFI (TCGA)
low (n=57)
high (n=19)
% Patients
HR = 0.9025
p = 0.7628
HR = 1.1493
p = 0.701
HR = 2.7
p = 0.0032
HR = 1.2391
p = 0.5148
Progression-free survival (days)

Figure S1. CHEK1 is an ERKi Sensitizers and Essential for PDAC growth, Related to Figure 1
(A) KRAS and TP53 mutation status, and SCH772984 ERK inhibitor sensitivity of PDAC cell lines used in this study.
(B) Categories of genes targeted by the CRISPR-Cas9 druggable genome library and experimental scheme for the screen.
(C) Categories of genes targeted by the siRNA druggable genome library and the experimental scheme for the screen.
(D) Heat map for the top 50 genes that increased ERKi sensitivity for each cell line and ERKi treatment time point. Shown is the median value of the replicates, scale bar represents the LogP (RSA).
(E) Comparison of kinase median dependency scores extracted from PDAC cells in DepMap and median log_2 fold change from our druggable genome CRISPR screen.
(F) Comparison of kinase median dependency scores from the DepMap CRISPR and DepMap shRNA screens.
(G) PDAC cell growth following treatment with siRNAs targeting, non-silencing control (NS), *KRAS*, *ATM*, *ATR*, or *CHEK1*. Viable cells were quantitated at days 1 and 5 following siRNA treatment. Statistical significance was evaluated using one-way ANOVA and Dunnett's multiple comparison test.
(H) Cell lysates were collected 72 h following siRNA treatment and evaluated for the indicated proteins by immunoblotting. A representative blot from two biological replicates is shown.
(I) TCGA pancreatic cancer patient data were analyzed for mRNA expression levels of the indicated DDR genes.
(J) Kaplan-Meier survival curves were determined from TCGA pancreatic cancer patient mRNA expression data and compared between the top and bottom quartiles based on expression of the indicated DDR gene.

Figure S2

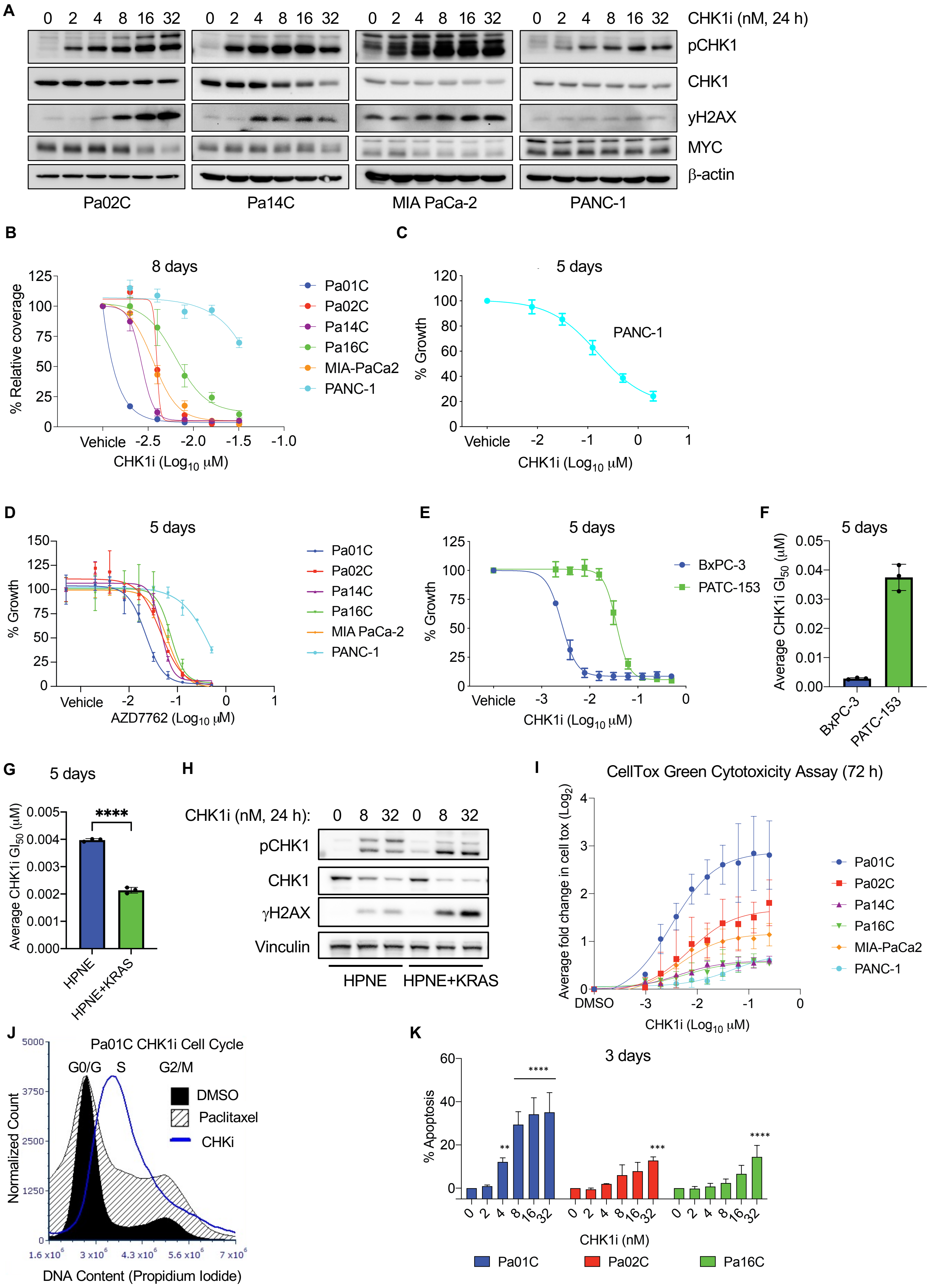
A
CHK1i (nM, 24 h)
0 2 4 8 16 32
pCHK1
CHK1
yH2AX
MYC
β-actin
Pa02C
Pa14C
MIA PaCa-2
PANC-1
B
8 days
% Relative coverage
CHK1i (Log10 µM)
Vehicle -2.5 -2.0 -1.5 -1.0
Pa01C
Pa02C
Pa14C
Pa16C
MIA-PaCa2
PANC-1
C
5 days
% Growth
PANC-1
CHK1i (Log10 µM)
D
5 days
% Growth
AZD7762 (Log10 µM)
Pa01C
Pa02C
Pa14C
Pa16C
MIA PaCa-2
PANC-1
E
5 days
% Growth
CHK1i (Log10 µM)
BxPC-3
PATC-153
F
5 days
Average CHK1i GI50 (µM)
BxPC-3
PATC-153
G
5 days
Average CHK1i GI50 (µM)
****
HPNE
HPNE+KRAS
H
CHK1i (nM, 24 h):
0 8 32 0 8 32
pCHK1
CHK1
γH2AX
Vinculin
HPNE
HPNE+KRAS
I
CellTox Green Cytotoxicity Assay (72 h)
Average fold change in cell tox (Log2)
DMSO
CHK1i (Log10 µM)
Pa01C
Pa02C
Pa14C
Pa16C
MIA-PaCa2
PANC-1
J
Pa01C CHK1i Cell Cycle
G0/G S G2/M
DMSO
Paclitaxel
CHKi
Normalized Count
DNA Content (Propidium Iodide)
K
3 days
% Apoptosis
CHK1i (nM)
Pa01C
Pa02C
Pa16C

Figure S2: CHK1i Blocks PDAC Growth and Induces S-phase Arrest and Apoptosis, Related to Figure 2

(A) The indicated cell lines were treated with CHK1i for 24 h and cell lysates were collected and probed for the indicated proteins.

(B) Quantification of mean % cell coverage and standard deviation from four biological replicates of clonogenic assays. The % coverage was quantified using masked images via FIJI. Example images from a representative replicate are shown in Figure 2B.

(C) Growth of CHK1-resistant PANC-1 cells was evaluated by live cell counting following CHK1i treatment for 5 days. Graph shows mean and standard deviation of biological triplicates; accompanies Figures 2C and 2D.

(D) Growth of PDAC cell lines was evaluated by live cell counting following treatment with additional CHK1i, AZD7762, for 5 days. The graph shows the mean and standard deviation of biological duplicates.

(E-F) Growth of KRAS wild-type PDAC cells following treatment with CHK1i, for 5 days.

(G) Growth of matched pairs of HPNE cells stably infected with empty vector or encoding $KRAS^{G12D}$ was evaluated by live cell counting following 5-day treatment with CHK1i. The graphs show the mean GI_{50} for each cell line and standard deviation of biological triplicates. Statistical significance was evaluated by unpaired t-test analysis; ****$p < 0.0001$.

(H) HPNE and HPNE($KRAS^{G12D}$) cells were treated with CHK1i for 24 h at the indicated doses. Cell lysates were collected and proteins were evaluated by immunoblot analyses.

(I) The average relative amounts of cell death were determined via CellTox Green staining and fluorescence levels at 72 h post CHK1i. Graphs represent the mean $\log_2$ fold change in CellTox Green over DMSO from three to four biological replicates and the corresponding standard deviation.

(J) CHK1i causes S phase accumulation in sensitive cell lines. The schematic shows a representative cell cycle fit of Pa01C cells treated with DMSO, paclitaxel, and CHK1i (8 nM) for 24 h.

(K) Cells were treated with CHK1i for 3 days and apoptosis levels were evaluated via FITC labeled Annexin V and flow cytometry. Graphs represent the mean of three biological replicates. Statistical significance was evaluated using one-way ANOVA and Dunnett's multiple comparison test; **$p < 0.01$, ***$p < 0.001$, ****$p < 0.0001$.

Figure S3

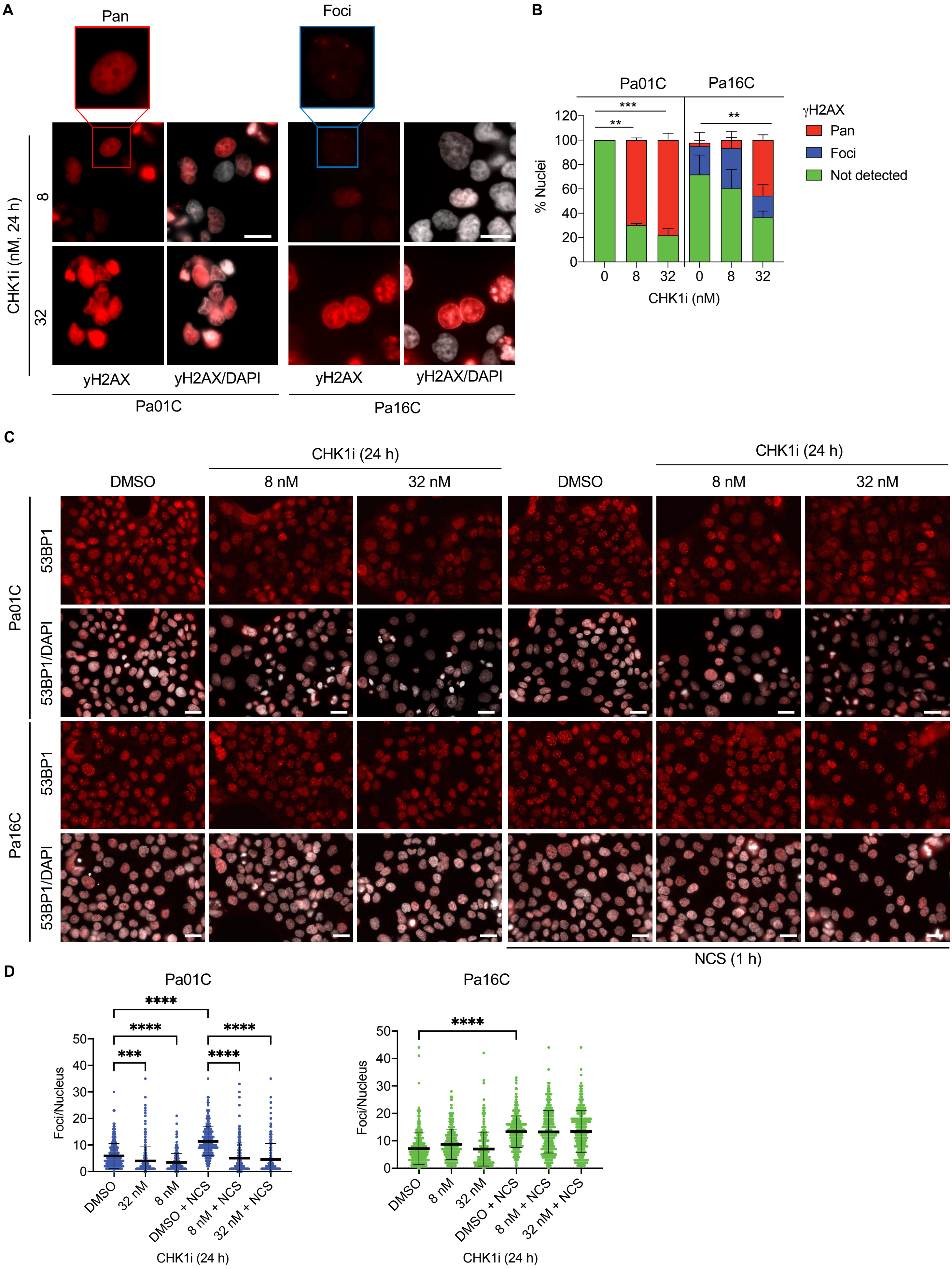
A
Pan
Foci
CHK1i (nM, 24 h)
8
32
yH2AX
yH2AX/DAPI
yH2AX
yH2AX/DAPI
Pa01C
Pa16C
B
Pa01C
Pa16C
% Nuclei
0
20
40
60
80
100
120
***
**
**
0
8
32
0
8
32
CHK1i (nM)
γH2AX
Pan
Foci
Not detected
C
CHK1i (24 h)
CHK1i (24 h)
DMSO
8 nM
32 nM
DMSO
8 nM
32 nM
Pa01C
Pa16C
53BP1
53BP1/DAPI
53BP1
53BP1/DAPI
NCS (1 h)
D
Pa01C
Pa16C
Foci/Nucleus
0
10
20
30
40
50
****
****
***
****
****
DMSO
32 nM
8 nM
DMSO + NCS
8 nM + NCS
32 nM + NCS
CHK1i (24 h)
Foci/Nucleus
****
DMSO
8 nM
32 nM
DMSO + NCS
8 nM + NCS
32 nM + NCS
CHK1i (24 h)

Figure S3: CHK1i promotes DNA damage and Loss of 53BP1 Mediated Repair, Related to Figure 3
(A-B) PDAC cells were treated with CHK1i at the indicated doses for 24 h, then fixed, stained for γH2AX and the nuclear marker DAPI, and imaged via wide-field microscopy, scale bar, 25 µm. (A) Representative images showing γH2AX pan-nuclear distribution and foci following CHK1i. (B) The mean of % nuclei for each γH2AX phenotype was calculated from three biological replicates. Error bars represent the standard deviation. Significance was evaluated using one-way ANOVA with Dunnett's multiple comparison test; **p < 0.01, ***p < 0.001.
(C) Representative images showing 53BP1 distribution following CHK1i treatment (24 h) of control or neocarzinostatin (NCS) treated (1 h) cultures. Cells were then fixed and stained for endogenous 53BP1 and the fluorescent DNA stain DAPI to visualize nuclei, and imaged using wide-field microscopy. Scale bar, 25 µm.
(D) Quantification of 53BP1 foci per nuclei from cells treated as shown panel (C). The mean and standard deviation are shown. Each dot represents one nucleus (collected from 2 independent biological replicates). Statistical significance was evaluated using one-way ANOVA with Sidak's multiple comparison test; ***p < 0.001, ****p < 0.0001.

Figure S4

A

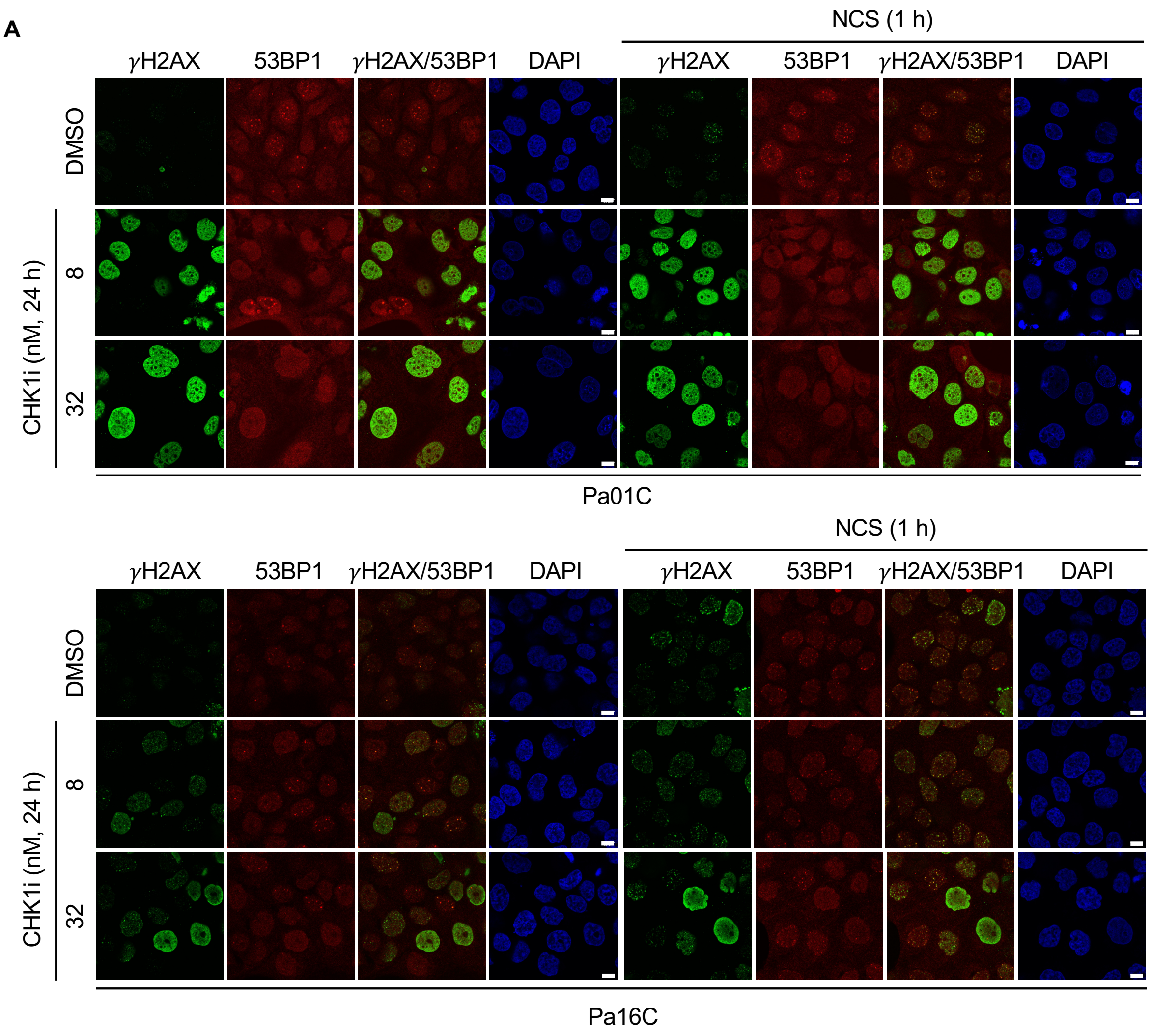

B

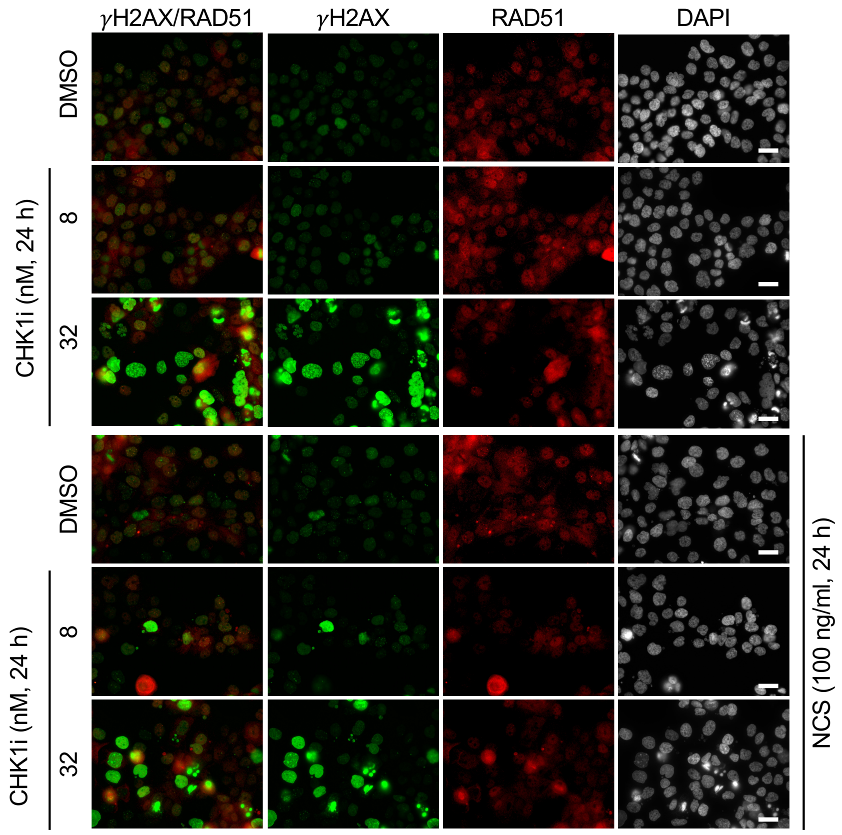

C

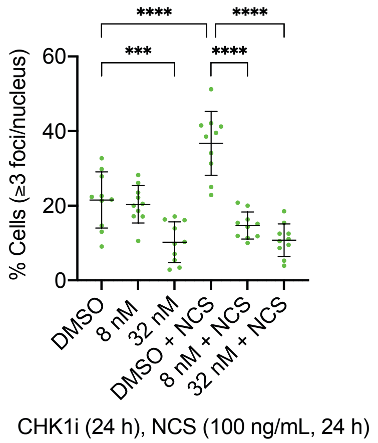

Figure S4: CHK1i promotes DNA damage and Loss of 53BP1 Mediated Repair, Related to Figure 3
(A) PDAC cells were treated with CHK1i at the indicated doses for 24 h, and/or the irradiation mimic, neocarzinostatin (NCS) for 1 h at 100 ng/ml. then fixed, stained for endogenous 53BP1, γH2AX, and the nuclear marker DAPI, and imaged via confocal microscopy. Scale bar, 10 µm.
(B) Representative images showing RAD51 and γH2AX distribution Control or NCS-treated (100 ng/ml) PDAC cells were treated with CHK1i at the indicated concentrations for 24 h, then fixed and stained for endogenous RAD51, γH2AX, and nuclei (DAPI), and imaged via wide-field microscopy. Scale bar, 25 µm.
(C) Quantification of percent nuclei containing RAD51 foci $\geq$ 3 per nucleus in Pa16C cells following each treatment shown in panel (F). Each dot represents one field of view representing > 400 nuclei and imaged from biological duplicates. Mean and standard deviation are shown. Statistical significance was evaluated using one-way ANOVA with Sidak's multiple comparison test; ***p < 0.001, ****p < 0.0001.

Figure S5

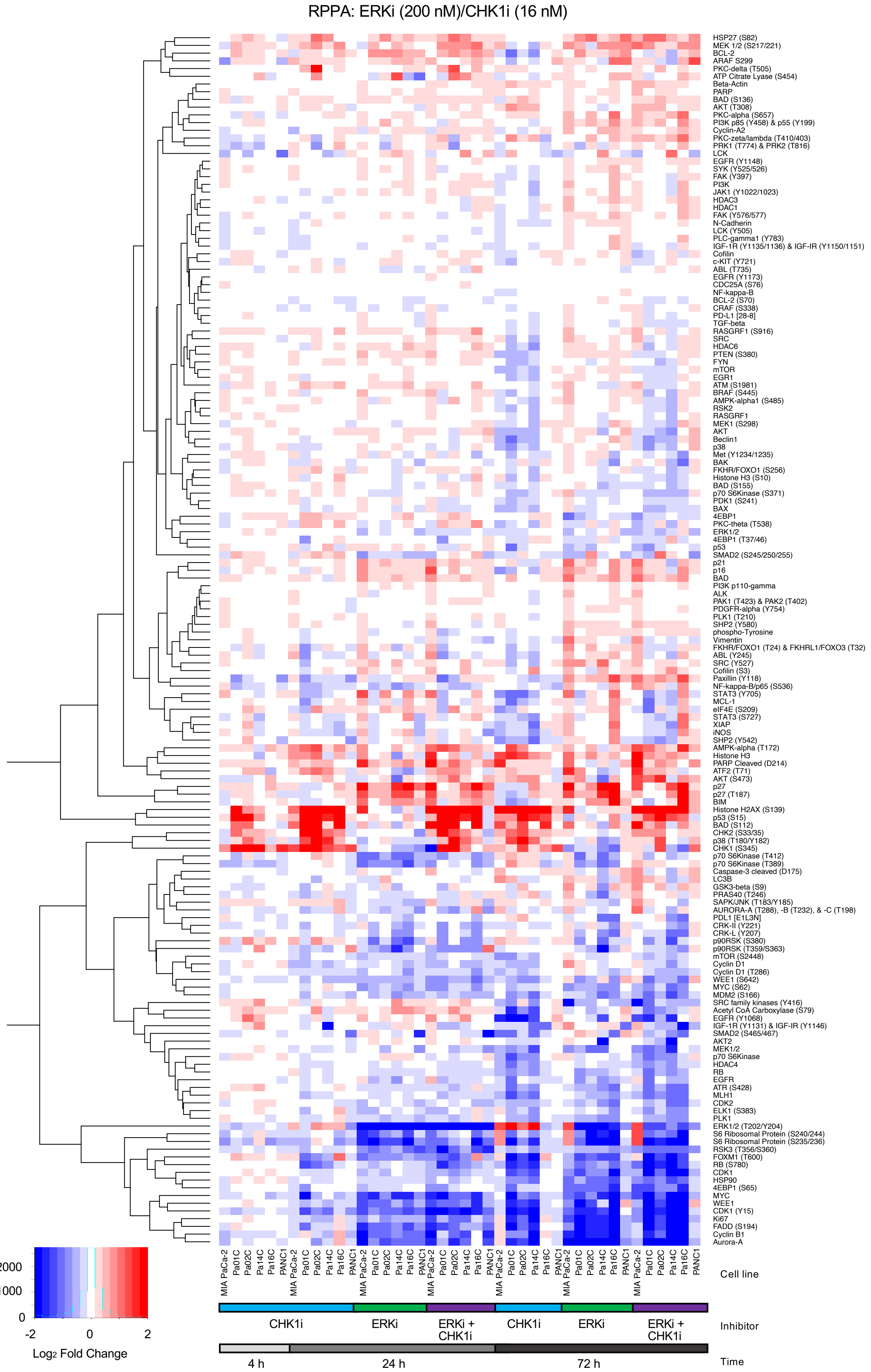
RPPA: ERKi (200 nM)/CHK1i (16 nM)
HSP27 (S82)
MEK 1/2 (S217/221)
BCL-2
ARAF S299
PKC-delta (T505)
ATP Citrate Lyase (S454)
Beta-Actin
PARP
BAD (S136)
AKT (T308)
PKC-alpha (S657)
PI3K p85 (Y458) & p55 (Y199)
Cyclin-A2
PKC-zeta/lambda (T410/403)
PRK1 (T774) & PRK2 (T816)
LCK
EGFR (Y1148)
SYK (Y525/526)
FAK (Y397)
PI3K
JAK1 (Y1022/1023)
HDAC3
HDAC1
FAK (Y576/577)
N-Cadherin
LCK (Y505)
PLC-gamma1 (Y783)
IGF-1R (Y1135/1136) & IGF-IR (Y1150/1151)
Cofilin
c-KIT (Y721)
ABL (T735)
EGFR (Y1173)
CDC25A (S76)
NF-kappa-B
BCL-2 (S70)
CRAF (S338)
PD-L1 [28-8]
TGF-beta
RASGRF1 (S916)
SRC
HDAC6
PTEN (S380)
FYN
mTOR
EGR1
ATM (S1981)
BRAF (S445)
AMPK-alpha1 (S485)
RSK2
RASGRF1
MEK1 (S298)
AKT
Beclin1
p38
Met (Y1234/1235)
BAK
FKHR/FOXO1 (S256)
Histone H3 (S10)
BAD (S155)
p70 S6Kinase (S371)
PDK1 (S241)
BAX
4EBP1
PKC-theta (T538)
ERK1/2
4EBP1 (T37/46)
p53
SMAD2 (S245/250/255)
p21
p16
BAD
PI3K p110-gamma
ALK
PAK1 (T423) & PAK2 (T402)
PDGFR-alpha (Y754)
PLK1 (T210)
SHP2 (Y580)
phospho-Tyrosine
Vimentin
FKHR/FOXO1 (T24) & FKHRL1/FOXO3 (T32)
ABL (Y245)
SRC (Y527)
Cofilin (S3)
Paxillin (Y118)
NF-kappa-B/p65 (S536)
STAT3 (Y705)
MCL-1
eIF4E (S209)
STAT3 (S727)
XIAP
iNOS
SHP2 (Y542)
AMPK-alpha (T172)
Histone H3
PARP Cleaved (D214)
ATF2 (T71)
AKT (S473)
p27
p27 (T187)
BIM
Histone H2AX (S139)
p53 (S15)
BAD (S112)
CHK2 (S33/35)
p38 (T180/Y182)
CHK1 (S345)
p70 S6Kinase (T412)
p70 S6Kinase (T389)
Caspase-3 cleaved (D175)
LC3B
GSK3-beta (S9)
PRAS40 (T246)
SAPK/JNK (T183/Y185)
AURORA-A (T288), -B (T232), & -C (T198)
PDL1 [E1L3N]
CRK-II (Y221)
CRK-L (Y207)
p90RSK (S380)
p90RSK (T359/S363)
mTOR (S2448)
Cyclin D1
Cyclin D1 (T286)
WEE1 (S642)
MYC (S62)
MDM2 (S166)
SRC family kinases (Y416)
Acetyl CoA Carboxylase (S79)
EGFR (Y1068)
IGF-1R (Y1131) & IGF-IR (Y1146)
SMAD2 (S465/467)
AKT2
MEK1/2
p70 S6Kinase
HDAC4
RB
EGFR
ATR (S428)
MLH1
CDK2
ELK1 (S383)
PLK1
ERK1/2 (T202/Y204)
S6 Ribosomal Protein (S240/244)
S6 Ribosomal Protein (S235/236)
RSK3 (T356/S360)
FOXM1 (T600)
RB (S780)
CDK1
HSP90
4EBP1 (S65)
MYC
WEE1
CDK1 (Y15)
Ki67
FADD (S194)
Cyclin B1
Aurora-A
2000
1000
0
-2
0
2
Log2 Fold Change
MIA PaCa-2
Pa01C
Pa02C
Pa14C
Pa16C
PANC1
Cell line
CHK1i
ERKi
ERKi + CHK1i
CHK1i
ERKi
ERKi + CHK1i
Inhibitor
4 h
24 h
72 h
Time

Figure S5: CHK1i promotes DNA damage and Loss of 53BP1 Mediated Repair, Related to Figure 3
Cells were treated with either CHK1i, ERKi, or ERKi+CHK1i for the indicated times. Cell lysates were collected and analyzed via RPPA using 162 antibodies. A complete list of antibodies is in Supplemental Table 1. The heatmap shows the relative median change upon inhibitor treatment compared to DMSO control for each antibody.

Figure S6

A

Top UP Regulated Proteins / Top DOWN Regulated Proteins

4 h	Protein	Mean FC log2	Pathway	4 h	Protein	Mean FC log2	Pathway
1	CHK1 (S345)	1.58	DNA damage Repair/Cell Cycle	1	p70 S6Kinase (T389)	-0.43	PI3K-MTOR Signaling
2	p53 (S15)	1.26	DNA damage Repair/Cell Cycle	2	LCK	-0.41	RTK Signaling
3	Histone H2AX (S139)	0.76	DNA damage Repair	3	S6 Ribosomal Protein (S235/236)	-0.40	PI3K-MTOR Signaling
4	EGFR (Y1068)	0.38	RTK Signaling	4	ERK1/2 (T202/Y204)	-0.32	MAPK Signaling
5	CHK2 (S33/35)	0.37	DNA damage Repair/Cell Cycle	5	CDK1 (Y15)	-0.27	Cell Cycle
6	BAD (S112)	0.36	Apoptosis	6	MYC	-0.25	MAPK/PI3K-MTOR/Cell Cycle/Metabolism/RTK/Transcription
7	p90-RSK (S380)	0.31	MAPK Signaling	7	Paxillin (Y118)	-0.24	Cytoskeleton
8	SRC family (Y416)	0.31	MAPK/PI3K-MTOR Signaling	8	S6 Ribosomal Protein (S240/244)	-0.24	PI3K-MTOR Signaling
9	Acetyl CoA Carboxylase (S79)	0.29	Metabolism	9	Cyclin B1	-0.22	Cell Cycle
10	BCL-2	0.27	Apoptosis	10	PRK1 (T774) & PRK2 (T816)	-0.21	Cytoskeleton
11	PARP-cleaved (D214)	0.24	Apoptosis	11	NF-kappa-B/p65 (S536)	-0.20	Transcription
12	IGF-1R (Y1131) & IGF-IR (Y1146)	0.24	RTK Signaling	12	WEE1 (S642)	-0.19	DNA damage Repair/Cell Cycle
13	p38 (T180/Y182)	0.22	MAPK Signaling	13	PKC-zeta/lambda (T410/403)	-0.17	PI3K-MTOR Signaling
14	p27	0.22	DNA damage Repair/Cell Cycle	14	RSK3 (T356/S360)	-0.16	MAPK Signaling
15	FOXM1 (T600)	0.22	DNA damage Repair/Cell Cycle/Transcription	15	AURORA-A	-0.15	DNA damage Repair/Cell Cycle
16	MEK1/2 (S217/221)	0.21	MAPK Signaling	16	EGFR	-0.14	RTK Signaling
17	AMPK-alpha (T172)	0.20	PI3K-MTOR Signaling/Metabolism	17	p70 S6Kinase (T412)	-0.13	PI3K-MTOR Signaling
18	ATR (S428)	0.19	DNA damage Repair/Cell Cycle	18	AURORA-A (T288), -B (T232), & -C (T198)	-0.13	DNA damage Repair/Cell Cycle
19	HSP27 (S82)	0.18	Apoptosis	19	LC3B	-0.12	Metabolism
20	eIF4E (S209)	0.17	Transcription	20	AKT (S473)	-0.12	PI3K-MTOR Signaling
**24 h**	**Protein**	**Mean FC log2**	**Pathway**	**24 h**	**Protein**	**Mean FC log2**	**Pathway**
1	Histone H2AX (S139)	2.30	DNA damage Repair/Cell Cycle	1	CDK1 (Y15)	-0.74	Cell Cycle
2	p53 (S15)	1.92	DNA damage Repair/Cell Cycle	2	RB (S780)	-0.60	Cell Cycle
3	CHK1 (S345)	1.75	DNA damage Repair/Cell Cycle	3	MYC	-0.55	MAPK/PI3K-MTOR/Cell Cycle/Metabolism/RTK/Transcription
4	BAD (S112)	1.50	Apoptosis	4	Paxillin (Y118)	-0.37	Cytoskeleton
5	p38 (T180/Y182)	0.99	MAPK Signaling	5	NF-kappa-B/p65 (S536)	-0.37	Transcription
6	CHK2 (S33/35)	0.97	DNA damage Repair/Cell Cycle	6	Cyclin D1	-0.36	Cell Cycle
7	AMPK-alpha (T172)	0.75	PI3K-MTOR Signaling/Metabolism	7	WEE1	-0.35	DNA damage Repair/Cell Cycle
8	Histone H3	0.45	Transcription	8	WEE1 (S642)	-0.33	DNA damage Repair/Cell Cycle
9	ATF2 (T71)	0.43	Transcription	9	S6 Ribosomal Protein (S235/236)	-0.32	PI3K-MTOR Signaling
10	MEK1/2 (S217/221)	0.43	MAPK Signaling	10	FOXM1 (T600)	-0.26	Cell Cycle/Transcription
11	PKC-delta (T505)	0.43	PI3K-MTOR Signaling	11	Cyclin D1 (T286)	-0.26	Cell Cycle
12	HSP27 (S82)	0.39	Apoptosis	12	FADD (S194)	-0.26	Apoptosis
13	PARP-cleaved (D214)	0.38	Apoptosis	13	MDM2 (S166)	-0.25	DNA damage Repair/Cell Cycle
14	p90-RSK (S380)	0.28	MAPK Signaling	14	iNOS	-0.25	Metabolism
15	4EBP1	0.26	PI3K-MTOR Signaling/Transcription	15	Ki67	-0.23	Cell Cycle
16	ATM (S1981)	0.25	DNA damage Repair/Cell Cycle	16	SMAD2 (S465/467)	-0.22	PI3K-MTOR Signaling
17	ARAF (S299)	0.24	MAPK Signaling	17	RSK3 (T356/S360)	-0.21	MAPK Signaling
18	SMAD2 (S245/250/255)	0.23	Apoptosis	18	XIAP	-0.19	Apoptosis
19	ERK1/2 (T202/Y204)	0.22	MAPK Signaling	19	HSP90	-0.18	MAPK Signaling
20	PKC-theta (T538)	0.22	PI3K-MTOR Signaling	20	MLH1	-0.18	DNA damage Repair
**72 h**	**Protein**	**Mean FC log2**	**Pathway**	**72 h**	**Protein**	**Mean FC log2**	**Pathway**
1	Histone H2AX (S139)	3.11	DNA damage Repair/Cell Cycle	1	CDK1 (Y15)	-1.26	Cell Cycle
2	p53 (S15)	1.11	DNA damage Repair/Cell Cycle	2	EGFR (Y1068)	-1.06	RTK Signaling
3	ERK1/2 (T202/Y204)	0.96	MAPK Signaling	3	WEE1	-0.89	DNA damage Repair/Cell Cycle
4	Histone H3	0.91	Transcription	4	AURORA-A	-0.88	DNA damage Repair/Cell Cycle
5	p38 (T180/Y182)	0.82	MAPK Signaling	5	RB (S780)	-0.88	Cell Cycle
6	BAD (S112)	0.81	Apoptosis	6	FOXM1 (T600)	-0.73	Cell Cycle/Transcription
7	CHK1 (S345)	0.79	DNA damage Repair/Cell Cycle	7	S6 Ribosomal Protein (S235/236)	-0.72	PI3K-MTOR Signaling
8	CHK2 (S33/35)	0.69	DNA damage Repair/Cell Cycle	8	FADD (S194)	-0.71	Apoptosis
9	PARP-cleaved (D214)	0.45	Apoptosis	9	Ki67	-0.69	Cell Cycle
10	AMPK-alpha (T172)	0.37	PI3K-MTOR Signaling	10	MYC	-0.67	MAPK/PI3K-MTOR/Cell Cycle/Metabolism/RTK/Transcription
11	AKT (S473)	0.35	PI3K-MTOR Signaling	11	SMAD2 (S465/467)	-0.65	PI3K-MTOR Signaling
12	AKT (T308)	0.30	PI3K-MTOR Signaling	12	MLH1	-0.64	DNA damage Repair
13	Beta-Actin	0.25	Actin	13	STAT3 (Y705)	-0.64	MAPK Signaling
14	ATF2 (T71)	0.23	Transcription	14	Acetyl CoA Carboxylase (S79)	-0.62	Metabolism
15	PKC-zeta/lambda (T410/403)	0.23	PI3K-MTOR Signaling	15	CDK1	-0.61	Cell Cycle
16	p70 S6Kinase (T412)	0.20	PI3K-MTOR Signaling	16	p70 S6Kinase	-0.57	PI3K-MTOR Signaling
17	p90-RSK (S380)	0.18	MAPK Signaling	17	SHP2 (Y542)	-0.56	RTK Signaling
18	BAD	0.18	Apoptosis	18	Cyclin B1	-0.52	Cell Cycle
19	BAD (S136)	0.17	Apoptosis	19	MEK1/2	-0.49	MAPK Signaling
20	Caspase-3 cleaved (D175)	0.16	Apoptosis	20	HSP90	-0.47	MAPK Signaling

UP at all time points | UP and DOWN at different time points | DOWN at all time points

B

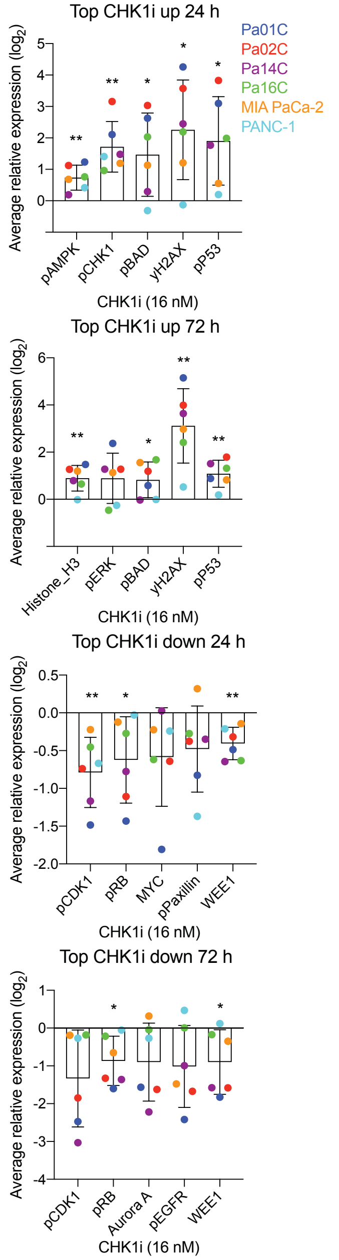

C

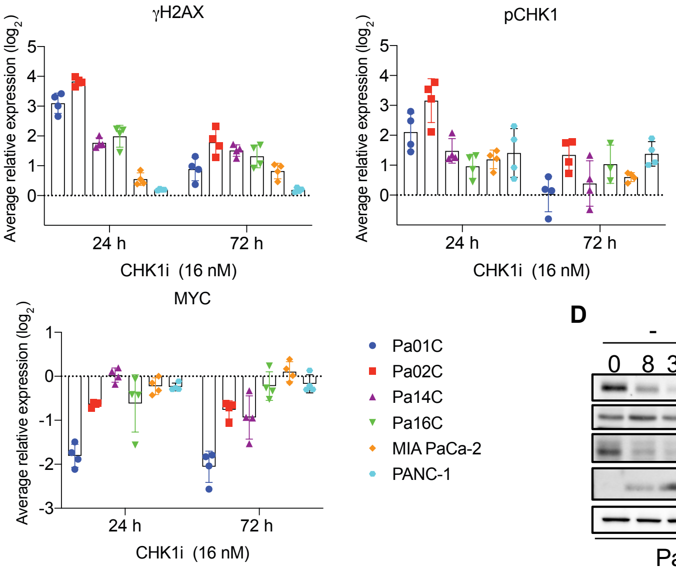

D

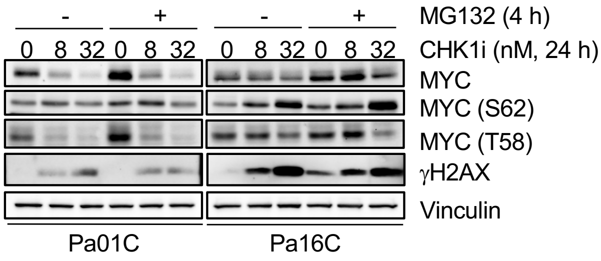

E

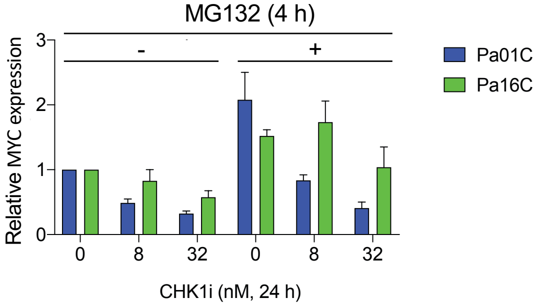

F

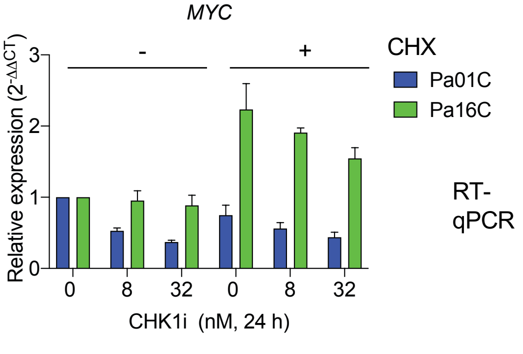

Figure S6: CHK1i promotes DNA damage and Loss of 53BP1 Mediated Repair, Related to Figure 3
(A) Top protein changes up and down following CHK1i, extracted from panel C. Changes listed in red represent the top upregulated proteins at all time points, blue reflects the top down-regulated changes at all times, and purple indicates proteins that switched from up to down or from down to up.
(B) The top five protein changes up and down following CHK1i, extracted from panel C. Each cell line point is the mean of four biological replicates analyzed by RPPA. Significance was evaluated using one-way ANOVA with Dunnett's multiple comparison test; *$p < 0.05$, **$p < 0.01$.
(C) Selected protein changes for each cell line at each time point relative to the respective DMSO treated control.
(D) Immunoblot analysis of MYC protein levels following CHK1i treatment with or without MG132. γH2AX was blotted to confirm CHK1i activity and vinculin served as a loading control.
(E) The mean relative expression of MYC protein quantified from three biological replicates, of which data in panel (E) is a representative example. Relative expression compares CHK1i treatment to vehicle (DMSO) control.
(F) qRT-PCR was used to determine *MYC* levels following CHK1i treatment with or without cycloheximide. Graph represents the mean from three biological replicates and their standard deviations.

Figure S7

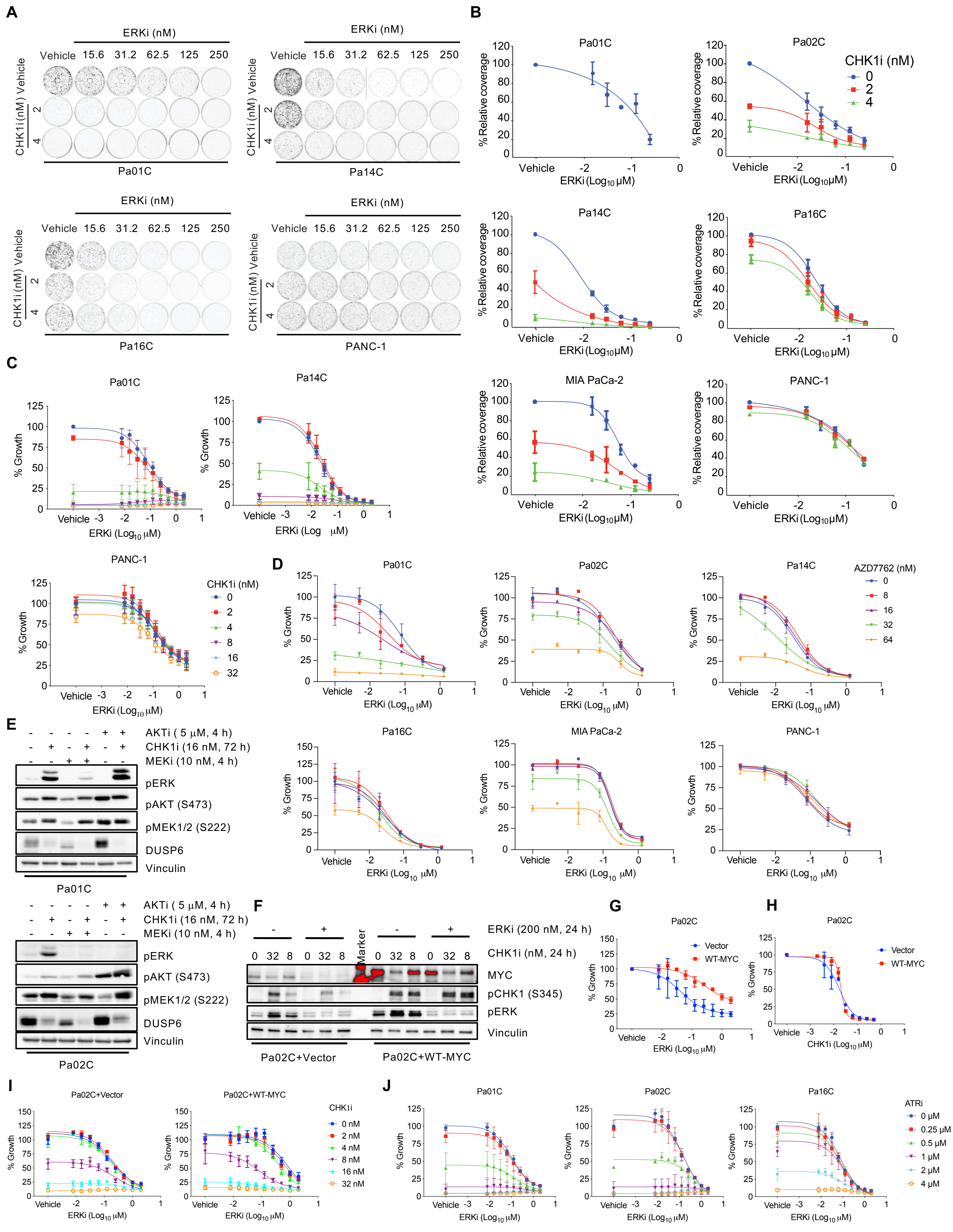

A
ERKi (nM)
Vehicle 15.6 31.2 62.5 125 250
CHK1i (nM) Vehicle 2 4
Pa01C
Pa14C
Pa16C
PANC-1
B
Pa01C
Pa02C
Pa14C
Pa16C
MIA PaCa-2
PANC-1
CHK1i (nM)
0
2
4
% Relative coverage
ERKi (Log10 μM)
C
Pa01C
Pa14C
PANC-1
% Growth
CHK1i (nM)
0
2
4
8
16
32
D
Pa01C
Pa02C
Pa14C
Pa16C
MIA PaCa-2
PANC-1
AZD7762 (nM)
0
8
16
32
64
E
AKTi (5 μM, 4 h)
CHK1i (16 nM, 72 h)
MEKi (10 nM, 4 h)
pERK
pAKT (S473)
pMEK1/2 (S222)
DUSP6
Vinculin
Pa01C
Pa02C
F
ERKi (200 nM, 24 h)
CHK1i (nM, 24 h)
Marker
MYC
pCHK1 (S345)
pERK
Vinculin
Pa02C+Vector
Pa02C+WT-MYC
G
Pa02C
Vector
WT-MYC
H
Pa02C
CHK1i (Log10 μM)
I
Pa02C+Vector
Pa02C+WT-MYC
CHK1i
0 nM
2 nM
4 nM
8 nM
16 nM
32 nM
J
Pa01C
Pa02C
Pa16C
ATRi
0 μM
0.25 μM
0.5 μM
1 μM
2 μM
4 μM

Figure S7: Concurrent CHK1i Treatment Enhances ERKi-mediated Growth Suppression and Apoptosis, Related to Figure 4

(A-B) Clonogenic growth assay to monitor growth suppression of PDAC cell lines treated for 8 days with the indicated inhibitors (A). Quantification of average % coverage and standard deviation from four biological replicates (B).

(C) Growth of PDAC cell lines was evaluated by live cell counting following CHK1i and/or ERKi treatment(s) for 5 days. Graphs show average and standard deviation of biological triplicates; accompanies Figure 4D.

(D) Cell growth was evaluated via live cell counting following CHK1 inhibition (AZD7762) and/or ERKi treatment(s) for 5 days. The graphs show average and standard deviation of biological duplicates.

(E) Cell lysates were collected following treatment with the indicated inhibitors, doses and treatment times are shown, and analyzed via immunoblotting.

(F-I) Pa02C cells stably expressing either WT-MYC or vector control were generated. (F) Cell lysates collected at 24 h post CHK1i and/or ERKi and analyzed for the indicated proteins via immunoblotting. (G-I) Cell growth was evaluated via live cell counting following ERKi (G), CHK1i (H), or CHK1i+ERKi (I). The graphs show the average and standard deviation of biological duplicates.

(J) Cell growth was evaluated via live cell counting following ATR inhibition (AZD6738) and/or ERKi treatment(s) for 5 days. The graphs show average and standard deviation of biological triplicates.

Figure S8

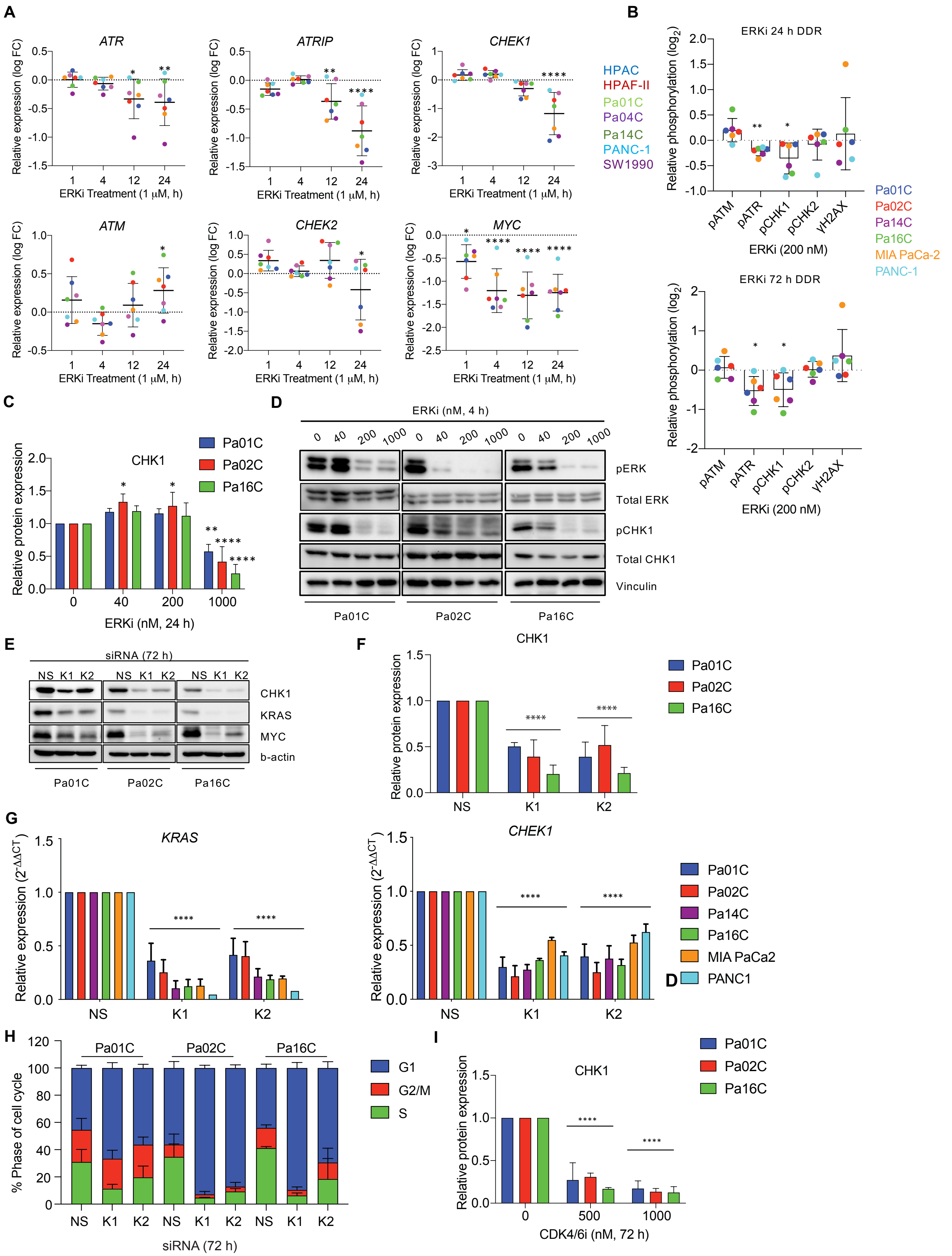

A
ATR
ATRIP
CHEK1
ATM
CHEK2
MYC
Relative expression (log FC)
ERKi Treatment (1 μM, h)
HPAC
HPAF-II
Pa01C
Pa04C
Pa14C
PANC-1
SW1990
B
ERKi 24 h DDR
ERKi 72 h DDR
Relative phosphorylation (log2)
pATM
pATR
pCHK1
pCHK2
γH2AX
ERKi (200 nM)
Pa01C
Pa02C
Pa14C
Pa16C
MIA PaCa-2
PANC-1
C
CHK1
Relative protein expression
ERKi (nM, 24 h)
Pa01C
Pa02C
Pa16C
D
ERKi (nM, 4 h)
0 40 200 1000
pERK
Total ERK
pCHK1
Total CHK1
Vinculin
Pa01C
Pa02C
Pa16C
E
siRNA (72 h)
NS K1 K2
CHK1
KRAS
MYC
b-actin
F
CHK1
Relative protein expression
NS
K1
K2
G
KRAS
CHEK1
Relative expression (2^-ΔΔCT)
Pa01C
Pa02C
Pa14C
Pa16C
MIA PaCa2
PANC1
H
% Phase of cell cycle
siRNA (72 h)
G1
G2/M
S
I
CHK1
CDK4/6i (nM, 72 h)

Figure S8: ERK Inhibition Decreases *CHEK1* Gene and Protein Expression by Causing G1 Cell Cycle Arrest, Related to Figure 5

(A-B) Cells were treated with 1 μM ERKi for the indicated amount of time. Statistical significance was determined via dispersion corrected, moderated *t*-tests as implemented in limma. RNA was collected and analyzed via RNA-seq. Each dot represents a cell line and red indicates a significant decrease in the average of the seven cell lines evaluated; *p < 0.05, **p < 0.01, ****p < 0.0001.

(B) Reverse Phase Protein Array (RPPA) was used to evaluate protein changes following ERKi (200 nM) in six different PDAC cell lines at 24 and 72 h. Graphs show the mean of the six PDAC cell lines for the selected protein changes. Significance was determined via a one-way ANOVA and Dunnett's multiple comparison test; *p < 0.05, **p < 0.01.

(C) Quantification of CHK1 protein levels following 24 h of ERKi. Representative images are shown in Figure 5C. The mean and standard deviation were calculated from three biological replicates. Significance was determined using one-way ANOVA and Dunnett's multiple comparison test; *p < 0.05, **p < 0.01, ****p < 0.0001.

(D) Cell lysates were collected after 4 h of ERKi and evaluated for the indicated proteins by immunoblotting. A representative blot from two biological replicates is shown.

(E-G) KRAS was depleted in cells using two independent siRNAs or non-targeted control siRNA (NS) for 72 h. Cell lysates (E-F) and RNA (G) were collected. Protein changes were evaluated via immunoblotting (E-F) and RNA levels via qRT-PCR (G). Graphs show the mean and standard deviations from biological triplicates. Significance was determined using one-way ANOVA and Dunnett's multiple comparison test; ****p < 0.0001. (H) Following KRAS depletion for 72 h, cell cycle status was evaluated using propidium iodide and flow cytometry. Graph shows the mean and standard deviations from biological triplicates.

(I) Cells were treated with palbociclib (CDK4/6i, 72 h) and cell lysates were collected and evaluated via immunoblotting. Representative blots are shown in Figure 5H. Graph represents the mean and standard deviation of biological triplicates. Significance was determined using one-way ANOVA and Dunnett's multiple comparison test; ****p < 0.0001.

Figure S9

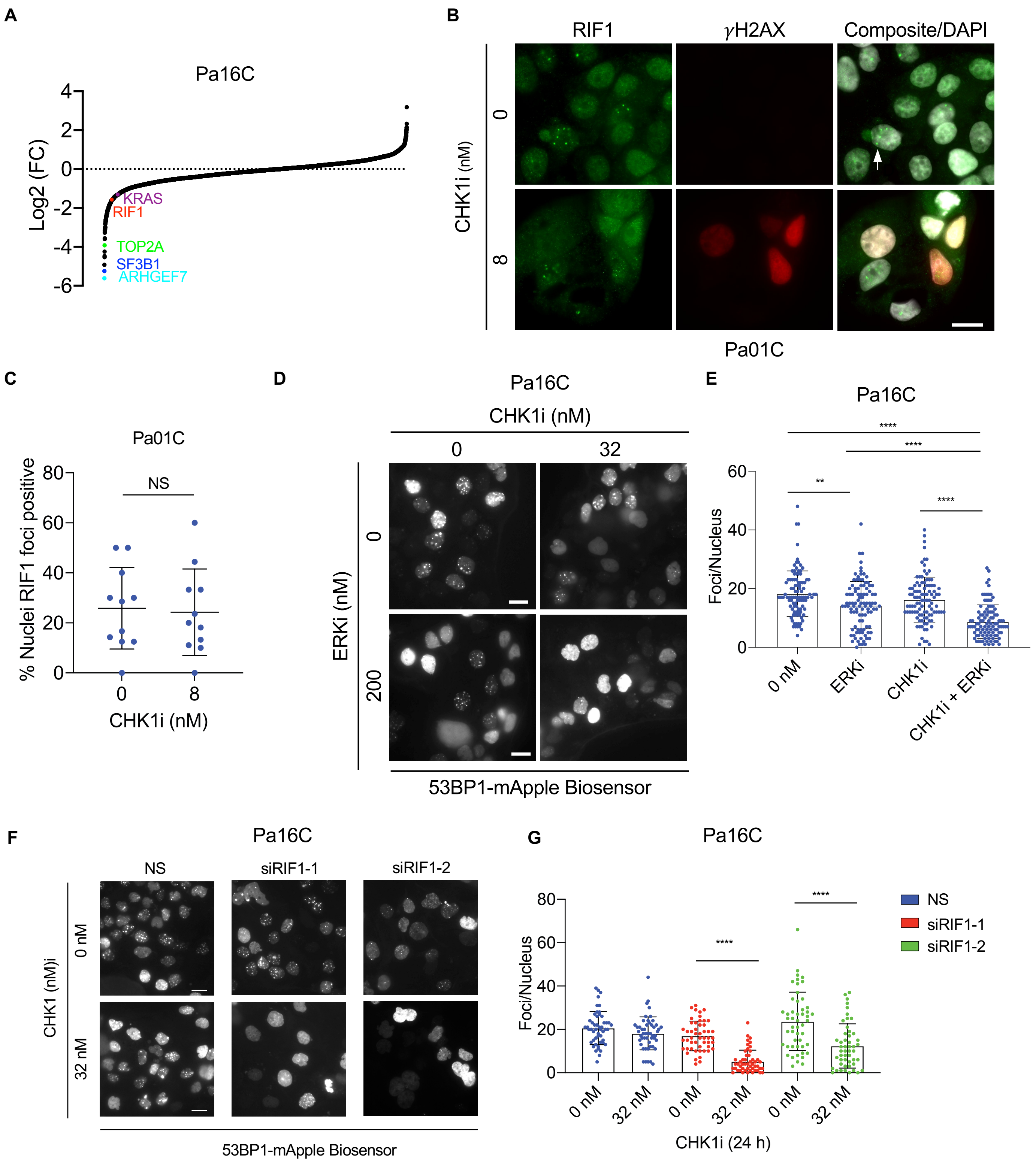
A
Pa16C
Log2 (FC)
4
2
0
-2
-4
-6
KRAS
RIF1
TOP2A
SF3B1
ARHGEF7
B
RIF1
γH2AX
Composite/DAPI
CHK1i (nM)
0
8
Pa01C
C
Pa01C
% Nuclei RIF1 foci positive
80
60
40
20
0
NS
0
8
CHK1i (nM)
D
Pa16C
CHK1i (nM)
0
32
ERKi (nM)
0
200
53BP1-mApple Biosensor
E
Pa16C
Foci/Nucleus
60
40
20
0
****
****
**
****
0 nM
ERKi
CHK1i
CHK1i + ERKi
F
Pa16C
NS
siRIF1-1
siRIF1-2
CHK1 (nM)i
0 nM
32 nM
53BP1-mApple Biosensor
G
Pa16C
Foci/Nucleus
80
60
40
20
0
****
****
NS
siRIF1-1
siRIF1-2
0 nM
32 nM
0 nM
32 nM
0 nM
32 nM
CHK1i (24 h)

Figure S9: Loss of RIF1 Increases Sensitivity to CHK1i, Related to Figure 6

(A) Identification of RIF1 via a CRISPR/Cas9 loss of function screen for CHK1i sensitizers among known/putative ERK substrates performed in Pa16C. The $\log_2$ fold change for all genes evaluated is shown, with *RIF1* and other top hits highlighted.

(B-C) Representative images of Pa01C cells treated with CHK1i for 24 h and evaluated for RIF1 (green) and γH2AX (red) via immunofluorescence. DAPI (white) identified cell nuclei; arrow marks a nucleus containing RIF foci. Scale bar, 25 μm.

(C) Quantification of percent nuclei containing RIF1 foci in Pa01C cells following each treatment. Each dot represents one field of view and imaged from biological duplicates. Mean and standard deviation are shown; treatments were compared via an unpaired *t*-test; **p < 0.01, ****p < 0.0001.

(D) Representative images of 53BP1-mApple-positive foci in Pa16C cells treated with ERKi and/or CHK1i for 24 h. Scale bar, 25 μm.

(E) Quantification of 53BP1-mApple-positve foci per nucleus from Pa16C cells treated as shown in (D). The mean and standard deviation are shown. Each dot represents one nucleus. Statistical significance was evaluated using two-way ANOVA with Tukey's multiple comparison test; **p < 0.01, ****p < 0.0001.

(F) Representative images of 53BP1 foci following reverse transfection with NS or two different siRNAs targeting *RIF1* for 72 h, with or without CHK1i for 24 h, performed in Pa16C cells. Scale bar, 25 μm.

(G) Quantification of trun53BP1-mApple foci per nuclei from cells treated as described in (F). The mean and standard deviation are shown. Each dot represents one nucleus. Statistical significance was evaluated using two-way ANOVA with Tukey's multiple comparison test.

Figure S10

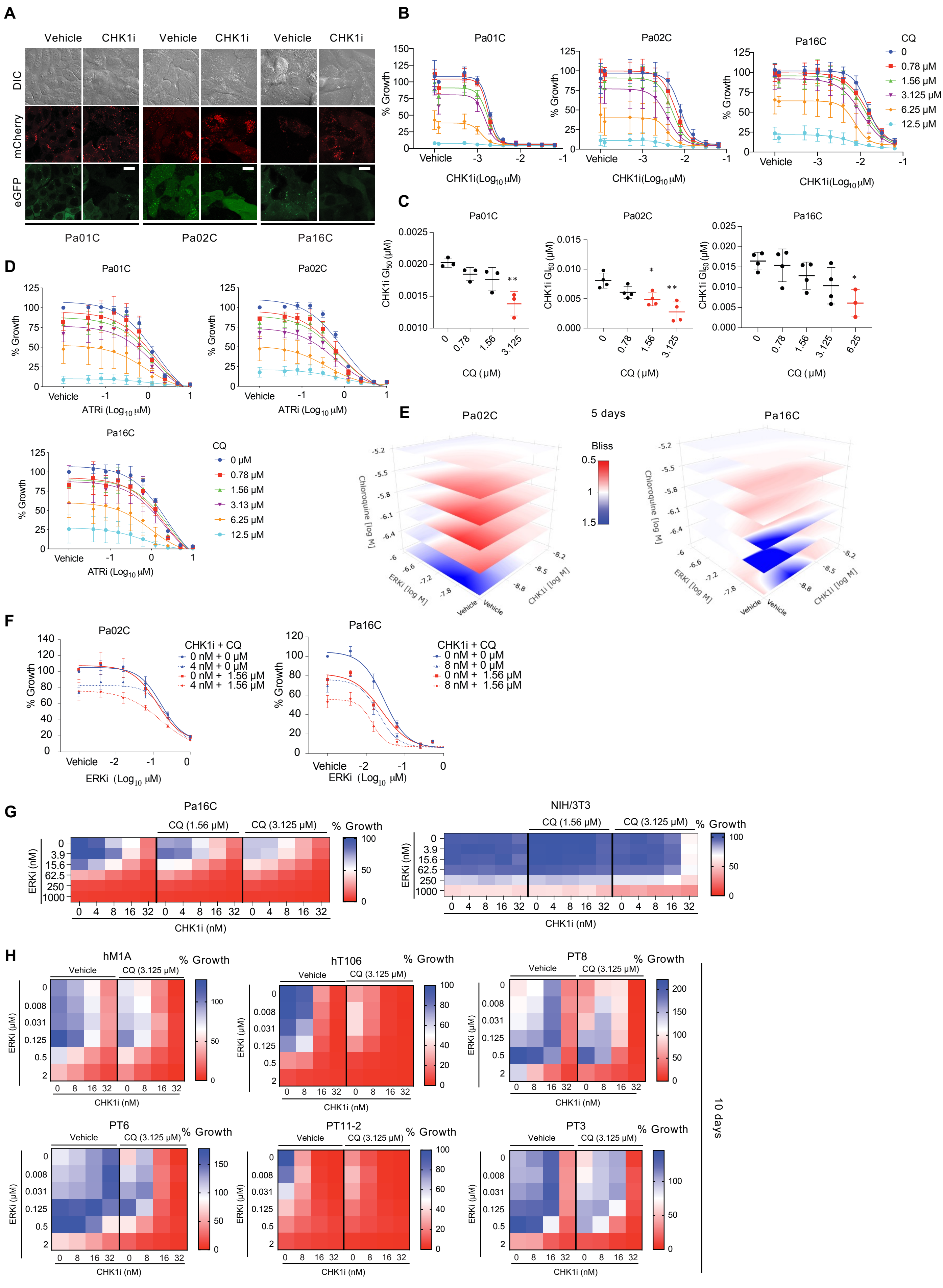

A
Vehicle
CHK1i
DIC
mCherry
eGFP
Pa01C
Pa02C
Pa16C
B
Pa01C
Pa02C
Pa16C
% Growth
CHK1i(Log10 µM)
CQ
0
0.78 µM
1.56 µM
3.125 µM
6.25 µM
12.5 µM
C
CHK1i GI50 (µM)
CQ (µM)
D
% Growth
ATRi (Log10 µM)
0 µM
3.13 µM
E
5 days
Bliss
Chloroquine [log M]
ERKi [log M]
CHK1i [log M]
Vehicle
F
CHK1i + CQ
0 nM + 0 µM
4 nM + 0 µM
0 nM + 1.56 µM
4 nM + 1.56 µM
8 nM + 0 µM
8 nM + 1.56 µM
ERKi (Log10 µM)
G
NIH/3T3
CQ (1.56 µM)
CQ (3.125 µM)
% Growth
ERKi (nM)
CHK1i (nM)
H
hM1A
hT106
PT8
PT6
PT11-2
PT3
ERKi (µM)
10 days

Figure S10: CHK1 Inhibition Induces Autophagy, Related to Figure 7

(A) Cell lines stably expressing the mCherry-EGFP-LC3B autophagic flux biosensor were imaged following treatment with CHK1i (24 h). Representative images for each cell line and treatment are shown. Scale bar, 25 μm.

(B) Cell growth was evaluated via live cell counting following treatment with CHK1i and CQ (5 days). Graph shows the mean and standard deviation from three to four biological replicates.

(C) The mean GI_{50} for the combination of CHK1i with CQ was determined from the growth assays performed in (B). Significant shifts are shown in red. One-way ANOVA with Dunnett's multiple comparison test was used to determine significance; *$p < 0.05$, **$p < 0.01$.

(D) Cell growth was evaluated by live cell counting following 5-day treatment with ATRi and CQ at the indicated concentrations. Graph shows the mean and standard deviation from three biological replicates.

(E-G) Cells were treated with the indicated concentrations of ERKi, CHK1i and/or chloroquine (CQ) for five days. (E) Bliss scores were determined based on the mean value of three biological replicates over a range of doses for each inhibitor. Synergy is indicated by red, antagonism by blue, and white reflects additivity.

(F) Graph shows growth inhibition of cells treated with ERKi alone or with CQ and/or CHK1i.

(G) Comparison 5-day growth inhibition of Pa16C and NIH/3T3 cells treated with CHK1i, ERKi and CQ at the indicated concentrations.

(H) Triple combinations were evaluated in patient-derived KRAS-mutant PDAC organoids. Organoids were treated (10 days) with the indicated concentrations of ERKi and CHK1i with or without CQ (3.125 μM). The median of three biological replicates for each treatment is shown. A shift from blue to red indicates reduction in organoid growth.
